# Disordered sodium alkoxides from powder data: crystal structures of sodium ethoxide, propoxide, butoxide and pentoxide, and some of their solvates

**DOI:** 10.1107/S205252062001584X

**Published:** 2021-01-21

**Authors:** Maurice Beske, Stephanie Cronje, Martin U. Schmidt, Lukas Tapmeyer

**Affiliations:** a Institut für Anorganische und Analytische Chemie, Goethe-Universität, Max-von-Laue-Strasse 7, 60438 Frankfurt am Main, Germany; b Department Chemie, Johannes Gutenberg-Universität Mainz, Duesbergweg 10-14, 55128 Mainz, Germany

**Keywords:** sodium alkoxide, powder data, solvate, isopropanol, Bärnighausen tree, PXRD

## Abstract

The crystal structures of sodium ethoxide (sodium ethano­late, NaOEt), sodium *n*-propoxide (sodium *n*-propano­late, NaO*^n^*Pr), sodium *n*-butoxide (sodium *n*-butano­late, NaO*^n^*Bu) and sodium *n*-pentoxide (sodium *n*-amylate, NaO*^n^*Am) were determined from powder X-ray diffraction data. NaOEt crystallizes in space group *P* 42_1_ *m*, with *Z* = 2, and the other alkoxides crystallize in *P*4/*nmm*, with *Z* = 2. To resolve space-group ambiguities, a Bärnighausen tree was set up, and Rietveld refinements were performed with different models. In all structures, the Na and O atoms form a quadratic net, with the alkyl groups pointing outwards on both sides (anti-PbO type). The alkyl groups are disordered. The disorder becomes even more pronounced with increasing chain length. Recrystallization from the corresponding alcohols yielded four sodium alkoxide solvates: sodium ethoxide ethanol disolvate (NaOEt·2EtOH), sodium *n*-propoxide *n*-propanol disolvate (NaO*^n^*Pr·2*^n^*PrOH), sodium isopropoxide iso­propanol pentasolvate (NaO*^i^*Pr·5*^i^*PrOH) and sodium *tert*-amylate *tert*-amyl alcohol monosolvate (NaO*^t^*Am·*^t^*AmOH, *^t^*Am = 2-methyl-2-butyl). Their crystal structures were determined by single-crystal X-ray diffraction. All these solvates form chain structures consisting of Na^+^, –O^−^ and –OH groups, encased by alkyl groups. The hydrogen-bond networks diverge widely among the solvate structures. The hydrogen-bond topology of the *^i^*PrOH network in NaO*^i^*Pr·5*^i^*PrOH shows branched hydrogen bonds and differs considerably from the networks in pure crystalline *^i^*PrOH.

## Abstract 2

The crystal structures of NaOEt, NaOPr, NaOBu and NaOAm (Am = amyl = pentyl) were determined from powder data. These compounds crystallize in an anti-PbO structure in the space groups *P* 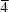
2_1_ *m* and *P*4/*nmm*. Additionally, solvates with the composition NaOEt·2EtOH, NaOPr·2PrOH, NaO*^i^*Pr·5*^i^*PrOH and NaO*^t^*Am·*^t^*AmOH were synthesized, and their structures were determined from single crystals. They form interesting chain structures of different compositions and topologies.

## 1. Introduction   

### 1.1. General   

Even today, there are simple chemical compounds for which the crystal structures are not known. The reasons for this deficiency in knowledge include synthetic difficulties, complex phase behaviour, instability in a vacuum and under an inert atmosphere, lack of single crystals, unusual or ambiguous space groups, and disorder. All these difficulties can be found in sodium alkoxides (sodium alcoholates) NaO*R* and their solvates NaO*R*·*x* *R*OH, with *R* being a lower alkyl group. In principle, these compounds can be easily prepared by the reaction of sodium with the corresponding alcohol. However, in practice, the synthesis of the pure phases presents some obstacles. For example, when sodium is reacted with ethanol and the ethanol excess is removed *in vacuo*, a white powder remains. This powder turns into a liquid within a few minutes under argon. Further evaporation under vacuum results in a powder, which again liquefies under argon. Finally, a white residue is obtained, which consists of a mixture of two to four different phases, including sodium ethoxide (NaOEt) and its ethanol disolvate NaOEt·2EtOH (Beske *et al.*, 2020 ▸). Phase-pure NaOEt is only obtained after a few hours of evaporation under vacuum at 50 °C. The initially formed solvate NaOEt·2EtOH decomposes under vacuum, and even under dry argon, and is stable only in the presence of ethanol vapour. Similar difficulties are observed for other sodium alkoxides (see below). Additionally, all the compounds are very sensitive to moisture.

In industry, as well as in the laboratory, sodium alkoxides are widely used as bases and as reagents in organic synthesis. This is not only true for NaOEt, but also for other alkoxides. For example, sodium *tert*-amylate (sodium 2-methyl-2-bu­tox­ide, NaO*^t^*Am) is used industrially on a multi-ton scale in the synthesis of diketo­pyrrolo­pyrrole pigments, which today are the most commonly used pigments for red car coatings (Hunger & Schmidt, 2018 ▸).

### 1.2. Historical notes on NaOEt   

Sodium ethoxide was synthesized as early as 1837 by Liebig (Liebig, 1837 ▸; Beske *et al.*, 2020 ▸). Since ethanol was considered the hydrate of ethyl ether (2EtOH ≙ Et_2_O·H_2_O), sodium ethoxide was regarded as an adduct of di­ethyl ether and sodium oxide, which actually corresponds to the correct stoichiometry: Et_2_O·Na_2_O ≙ 2NaOEt. Correspondingly, the name ‘Aethernatron’ (Geuther, 1868*a* ▸,*b* ▸) was used besides the names ‘Natriumalkoholat’ (Geuther, 1859 ▸) and ‘Natriumäthylat’ (Wanklyn, 1869 ▸).

Many years later, the crystal structure of sodium methoxide (NaOMe) was determined from powder X-ray diffraction (PXRD) data (Weiss, 1964 ▸). Surprisingly, the crystal structure of NaOEt was not determined, although it is isostructural with NaOMe. It would have been an easy task to index the powder pattern of NaOEt manually, because NaOEt crystallizes in the tetragonal crystal system, and the lattice parameters *a* and *b* of NaOEt are almost identical to those in NaOMe.

In 1976, the PXRD patterns of NaOEt and NaOEt·2EtOH were published in an article devoted to the thermal stability of alkali ethoxides (Blanchard *et al.*, 1976 ▸). Again, no attempt was made to index the powder data.

As much as 30 years later, the powder data of NaOEt and sodium *n*-propoxide (NaO*^n^*Pr) were indexed, but the crystal structures remained indeterminate (Chandran *et al.*, 2006 ▸). Finally, we determined the crystal structure of NaOEt from powder data, and of NaOEt·2EtOH from single-crystal data a few months ago. The crystal structures were recently briefly described in a chemical journal (Beske *et al.*, 2020 ▸), without any discussion on the ambiguity of the space group or of the crystal symmetry. Here, we report a full discussion of the ambiguities of the space group of NaOEt, including a Bärnighausen tree of the possible space groups and their subgroups.

### 1.3. Previous work on other alkoxides   

The first determined crystal structure of a sodium alkoxide was that of NaOMe (Weiss, 1964 ▸). NaOMe is isotypical to LiOMe (Wheatley, 1961 ▸) and crystallizes in a layer structure in the space group *P*4/*nmm*, with *Z* = 2.

Potassium methoxide, KOMe, crystallizes in the same space group type as NaOMe, but the structure is different: whereas the Na^+^ ions in NaOEt are coordinated to four O atoms, the K^+^ ions in KOMe are coordinated to five O atoms in a square-pyramidal geometry (Weiss, 1963 ▸; Weiss & Alsdorf, 1970 ▸). The O atom is surrounded by five K^+^ ions and the methyl group has a distorted octahedral geometry. A similar structure was found for partially hydrolysed NaOMe with the composition Na(OMe)_1–_ *_x_*(OH)*_x_*, with *x* ≈ 1/3 (Weiss, 1964 ▸).

Sodium *tert*-butoxide, NaO*^t^*Bu, exists in two polymorphic forms. Both structures were determined by single-crystal X-ray diffraction. One of the phases consists of hexamers and crystallizes in the space group *P*2_1_2_1_2_1_, with *Z* = 20, with five hexamers per asymmetric unit (Østreng *et al.*, 2014 ▸). The other phase contains a 1:1 mixture of hexamers and nonamers in the space group *R* 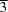, with *Z* = 6, with 90 formula units per unit cell (Greiser & Weiss, 1977 ▸; Davies *et al.*, 1982 ▸; Nekola *et al.*, 2002 ▸). Accordingly, both phases have quite large unit cells.

### 1.4. Solvates   

Sodium alkoxides can form solvates with their corresponding alcohols. Already in 1837 Liebig had prepared an ethanol solvate of NaOEt by the reaction of sodium with ethanol at 50 °C and subsequent cooling of the solution to room temperature, whereupon the mixture turned into a solid (Liebig, 1837 ▸). However, Liebig apparently did not recognize this precipitate as a solvate. In 1868, Scheitz determined the composition of this solvate as NaOEt·2EtOH (Geuther, 1868*a* ▸). This result was confirmed by Marsh (Geuther, 1868*b* ▸), whereas Wanklyn (1869 ▸) determined the composition to be NaOEt·3EtOH. In 1880, Frölich again found a composition of NaOEt·2EtOH using a different method (Geuther & Frölich, 1880 ▸). Lescoeur (1895 ▸) measured the vapour pressure during slow evaporation of a suspension of NaOEt in EtOH and observed that the vapour pressure did not change between compositions of NaOEt·1.7EtOH and nearly pure NaOEt, and thus concluded that the solvate had the composition NaOEt·2EtOH.

The crystal morphology of NaOEt·2EtOH was described as ‘völlig durchsichtige farblose nadelförmige Krystalle’ (fully transparent, colourless, needle-like crystals) (Geuther, 1868*a* ▸).

Geuther & Frölich (1880 ▸) also described a solvate with a com­position of NaO*^n^*Pr·2*^n^*PrOH. A *^t^*AmOH solvate of NaO*^t^*Am was mentioned by Friedrich *et al.* (1999 ▸), but no composition as given.

The solvates are thermally remarkably stable. NaOEt·2EtOH must be heated at ambient pressure to 200 °C and NaO*^n^*Pr·2*^n^*PrOH even to 220 °C before the pure solvent-free alkoxides are obtained (Geuther & Frölich, 1880 ▸). Solvent-free NaOEt is also quite stable. According to differential thermal analysis, the decomposition starts at 50 °C, but this decomposition is very slow and occurs over a large temperature range. Finally, at 310 °C the decomposition ‘adopts an explosive character’ (‘prendre un charactère explosif’; Blanchard *et al.*, 1976 ▸).

Crystals of the solvates of NaO*^n^*Pr, NaO*^i^*Pr and NaO*^t^*Am do form easily when sodium is reacted with the corresponding alcohols and the solution is subsequently carefully evaporated. However, no structure of any sodium alkoxide solvate was determined between 1837 and 2019 (Beske *et al.*, 2020 ▸). The reason might be the pronounced sensitivity of the crystals to moisture, air, vacuum and dry inert gas.

### 1.5. Work in this article   

In this article, we describe the synthesis, structure determination, crystal structure and disorder of NaOEt, NaO*^n^*Pr, NaO*^n^*Bu and NaO*^n^*Am, and of the solvates NaOEt·2EtOH, NaO*^n^*Pr·2^*n*^PrOH, NaO*^i^*Pr·5^i^PrOH and NaO*^t^*Am·*^t^*AmOH. The structures of the solvent-free compounds were determined by PXRD and the structures of the solvates by single-crystal X-ray analyses. In the cases of NaOEt and NaO*^n^*Pr, the space-group symmetry was ambiguous, and the corresponding symmetry relationships were elaborated using a Bärnighausen tree.

## 2. Experimental details   

### 2.1. Syntheses   

All synthetic procedures were performed under an argon atmosphere using Schlenk techniques. All alcohols, as well as toluene, were dried over sodium and freshly distilled.

#### 2.1.1. NaOEt   

0.34 g (15 mmol) of sodium were added to 10 ml (170 mmol) of ethanol. The mixture was allowed to react for 30 min at room temperature. The obtained solution was heated to 50 °C and the excess ethanol was removed under vacuum. The resulting solid product was evaporated at 50 °C under vacuum for 3 h, gently crushed with a glass rod and again evaporated for one additional hour under the same conditions. A phase-pure white powder of NaOEt was obtained.

#### 2.1.2. NaO*^n^*Pr, NaO*^n^*Bu and NaO*^n^*Am   

NaO*^n^*Pr, NaO*^n^*Bu and NaO*^n^*Am were synthesized in a similar manner to NaOEt. Details are given in the supporting information.

#### 2.1.3. NaOEt·2EtOH   

0.34 g of sodium (15 mmol) were added to 5.0 ml (85 mmol) of ethanol. After reacting for 30 min, a gel was obtained. This gel was stored for three months at room temperature, resulting in a pale-brown solution and colourless needles of NaOEt·2EtOH with a size of up to 1 mm.

#### 2.1.4. NaO*^n^*Pr·2*^n^*PrOH, NaO*^i^*Pr·5*^i^*PrOH and NaO*^t^*Am·*^t^*AmOH   

Syntheses and crystal growth of these compounds resembled the procedure used for NaOEt·2EtOH. Details are given in the supporting information.

### 2.2. Pre-characterization   

The stoichiometry of the solvent-free alkoxides was con­firmed by decomposition experiments with HCl, which verified their stoichiometry. Details are reported in the supporting information. For the solvates, this analysis could not be performed, because the solvates decomposed rapidly when removed from their alcoholic mother liquor.

### 2.3. Powder X-ray diffraction (PXRD)   

For the PXRD studies, the samples were sealed in glass capillaries with a 1.0 mm diameter. The PXRD patterns were measured in transmission mode on a Stoe Stadi-P diffrac­tometer equipped with a Ge(111) monochromator and a linear position-sensitive detector. The capillaries were spun during the measurements. All measurements were performed at room temperature, using Cu *K*α_1_ radiation (λ = 1.5406 Å), with a 2θ range of 2–100° (2–80° for NaOEt).

### 2.4. Structure determination from powder data   

The crystal structures of the solvent-free alkoxides NaOEt, NaO*^n^*Pr, NaO*^n^*Bu and NaO*^n^*Am were determined from PXRD data. The powder data were indexed with the program *DICVOL* (Boultif & Louër, 1991 ▸) within the program package *DASH* (David *et al.*, 2006 ▸). The structures were solved by the real-space method with simulated annealing using *DASH*. Subsequently, Rietveld refinements were performed using *TOPAS* (Coelho, 2018 ▸).

For all four compounds indexing led to a tetragonal unit cell with *Z* = 2. The systematic extinction indicated *P*4/*n*, *P*4/*nmm* and *P* 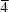
2_1_ *m* as possible space groups. The structures were successfully solved in *P*4/*nmm* and *P* 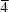
2_1_ *m*, using two fragments, an Na atom and a rigid alkoxide moiety. The Na atom was placed on the special position, which allowed a distorted tetrahedral coordination [Wyckoff position 2*a* (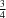, 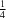, 0) in *P*4/*nmm* origin choice 2; 2*a* (0, 0, 0) in *P* 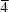
2_1_ *m*], as explained in §3.1. The alkoxide fragment was placed on a general position, with an occupancy of 0.125 (in *P*4/*nmm*) or 0.25 (in *P* 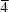
2_1_ *m*). In the resulting structures, the C atoms moved close to a site with ..*m* symmetry, and were subsequently placed on this site, resulting in an occupancy of 0.25 (in *P*4/*nmm*) or 0.5 (in *P* 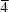
2_1_ *m*). In the Rietveld refinements of NaOEt, restraints were only necessary for the H atoms. For NaO*^n^*Pr, NaO*^n^*Bu and NaO*^n^*Am, additional restraints were applied to the O—C and C—C bond lengths, and to the O—C—C and C—C—C bond angles. All H atoms were refined using restraints on the bond lengths and angles with quite high weights. Further details of the Rietveld refinements are given in the supporting information.

Note that there are two different origin choices for *P*4/*nmm*. Origin choice 2 (origin on 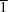) was used for the structure solution, due to the requirements of *DASH*. In contrast, the origin choice 1 (origin on 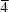, as in *P* 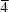
2_1_ *m*) was used for the Bärnighausen tree.

### 2.5. Single-crystal X-ray diffraction   

A single crystal of NaOEt·2EtOH was placed in a sealed glass capillary and data were collected at −38 (2) °C. Single crystals of NaO*^n^*Pr·2*^n^*PrOH and NaO*^i^*Pr·5*^i^*PrOH were mounted by freezing them in a drop of oil and their data col­lected under a cold nitro­gen stream at −100 (2) °C using an Oxford Cryosystems cryostream device. Crystals of NaO*^t^*Am·*^t^*AmOH were sealed in a glass capillary under paraffin oil (dried with Na) and their data collected at room temperature.

Single-crystal data were collected on a Bruker SMART APEX three-circle diffractometer equipped with an Incoatec IμS Cu microfocus source with mirror optics and an APEX II CCD detector. The software package *APEX3* (Bruker, 2015 ▸) was used for data collection and data reduction. The structures were solved by direct methods with *SHELXT* (Sheldrick, 2015*a* ▸) and refined with *SHELXL* (Sheldrick, 2015*b* ▸). All non-H atoms, except for disordered C atoms, were refined anisotropically. Disordered C atoms were refined isotropically. H atoms bonded to C atoms were treated with the riding model. In the case of NaOEt·2EtOH and NaO*^n^*Pr·2*^n^*PrOH, all OH protons could be located by Fourier synthesis. In NaO*^i^*Pr·5*^i^*PrOH, it was not possible to detect which of the four ligands coordinating to the Na^+^ ion is the *^i^*PrO^−^ anion, and which are the three *^i^*PrOH molecules. (The H atom could not be located, all Na—O bonds were of a similar length, all C—O bonds were of similar length, and in addition no decision could be made based on the size of the angles; furthermore, there is a twofold axis through the Na^+^ ion, hence there are always pairs of symmetrically equivalent ligands.) All O atoms of NaO*^i^*Pr·5*^i^*PrOH are part of a complex hydrogen-bond network, and obviously the H atoms are disordered within this network. Therefore, for each Na^+^ cation, H atoms with occupancies of 0.75 were placed at all four O atoms connected to the Na^+^ cation. For NaO*^t^*Am·*^t^*AmOH, the electron density indicates that the H atoms of the OH groups are located along hydrogen bonds. However, the limited data quality did not allow an unrestrained refinement of the positions of these H atoms. According to the charge compensation, the H atoms should be disordered, too.

The single crystals of NaO*^i^*Pr·5*^i^*PrOH are highly sensitive; they decompose within seconds except when they are kept in their mother liquor under an inert atmosphere. Therefore, only rather poor diffraction data could be obtained. Correspondingly, a large number of restraints had to be used in the refinement. The disordered C atoms were refined isotropically, with restraints on the C—C and C—O bond lengths. The ordered C atoms were refined anisotropically, but their anisotropic displacement parameters were restrained to be similar to those of neighbouring atoms.

## 3. Results and discussion   

### 3.1. Space group and disorder of sodium ethoxide (NaOEt)   

Sodium ethoxide is difficult to obtain as a pure phase. The reaction of sodium with ethanol, with subsequent evaporation at room temperature under vacuum or evaporation at the boiling point at ambient pressure, results in a mixture of two to four phases, including NaOEt and NaOEt·2EtOH. Evaporation under vacuum at 50 °C for several hours leads to phase-pure NaOEt. Nevertheless, most of our recorded powder patterns were contaminated by traces of other phases.

The powder pattern of NaOEt could be indexed with a tetragonal unit cell, with *a* = *b* = 6.2, *c* = 9.1 Å and *V* = 352 Å^3^. According to Hofmann’s volume increments (Hofmann, 2002 ▸), the unit-cell volume corresponds to *Z* = 4. The systematic extinctions pointed to the space group *P*4/*nbm*. Further experiments revealed that some of the weak peaks in the powder pattern were actually caused by foreign phases. The pattern of the phase-pure NaOEt could be indexed with a unit cell of half of the initial volume, with *a* = *b* = 4.41, *c* = 9.07 Å, α = β = γ = 90°, *V* = 176.4 Å^3^ and *Z* = 2.

The systematic extinctions lead to the extinction symbol *Pn*––, which corresponds to the space group *P*4/*n* or *P*4/*nmm* (Hahn, 2005 ▸). In *P*4/*nmm*, the structure could be solved without difficulty by the real-space method with simulated annealing using the program *DASH* (David *et al.*, 2006 ▸). The unit cell contains two formula units. In *P*4/*nmm* there are three different Wyckoff positions with a multiplicity of two: positions 2*a* and 2*b* with site symmetry 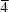 *m*2, and 2*c* with site symmetry 4*mm*. A tetrahedral coordination of the Na^+^ ion agrees with a 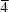 *m*2 site symmetry. Correspondingly, the Na^+^ ion was set at position 2*a*. A rigid C_2_H_5_O fragment was placed on the general position (16*k*) with an occupancy of 0.125. The best solution was found in about 10 out of 25 runs and had a good profile-χ^2^ value of 7.26. The O atom was found very close to the 4*mm* site (Wyckoff position 2*c*), hence it could be set at this site. The ethyl group is disordered around the 4*mm* site. The two C atoms could be situated on the general position (16*k*), resulting in eightfold disorder, or on mirror planes parallel to (100) and (010) (Wyckoff position 8*i*, site symmetry .*m*.), or on diagonal mirror planes (Wyckoff position 8*j*, site symmetry ..*m*), each with fourfold disorder.

**Figure 1:**
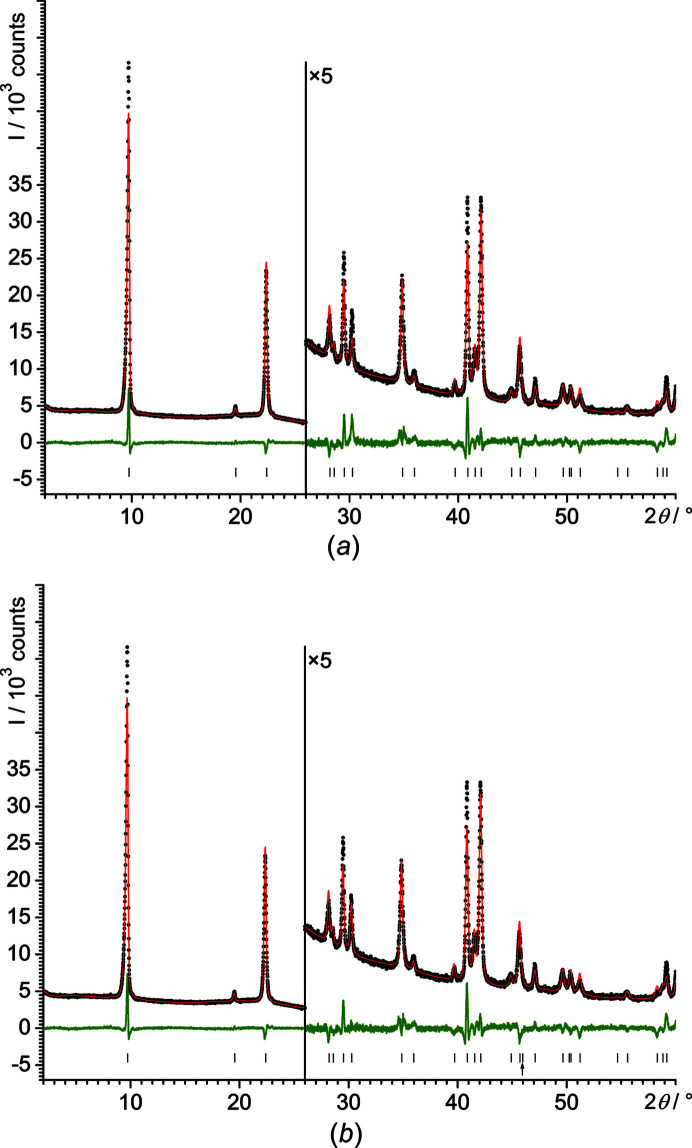
Rietveld plots of NaOEt performed in different space groups under identical conditions, *i.e.* (*a*) *P*4/*nmm* and (*b*) *P* 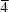
2_1_ *m*. Experimental data are shown as black dots and simulated data as a red line, with the difference curve in green below. The vertical tick marks denote the reflection positions. The small arrow in (*b*) denotes the 210 reflection, which is extinct in *P*4/*nmm*, but present in *P* 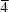
2_1_ *m*.

**Figure 2:**
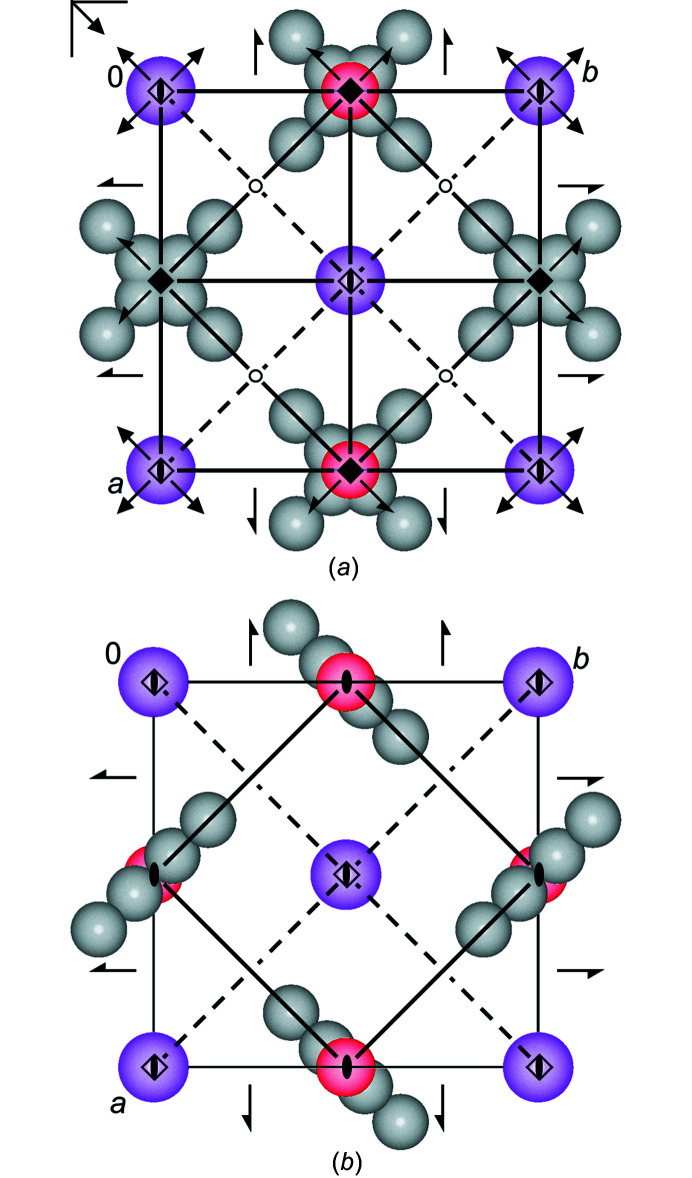
Structural models of NaOEt in (*a*) *P*4/*nmm* (origin choice 1) and (*b*) *P* 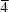
2_1_ *m*. Colour key: Na violet, O red and C grey (disordered). H atoms have been omitted for clarity. The view direction is [001]. The crystallographic symmetry elements are included.

The structure was refined by the Rietveld^1^ method (Loopstra & Rietveld, 1969 ▸) with *TOPAS*, with the C atoms on the general position (16*k*). During the refinement, the C atoms moved close to the diagonal mirror planes. Correspondingly, they were set to the sites 8*j* (..*m*). The refinements converged with good *R* values (Table 1 ▸) and smooth difference curves (Fig. 1 ▸ *a*). The ethyl groups are fourfold disordered around the fourfold axes, see Fig. 2 ▸(*a*). A corresponding structure was also found for lithium methoxide (LiOMe) (Wheatley, 1961 ▸) and sodium methoxide (NaOMe) (Weiss, 1964 ▸).

A symmetry analysis revealed that in the subgroup *P* 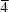
2_1_ *m* the ethyl groups would have a twofold disorder only. *P* 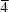
2_1_ *m* is a *translationengleiche* subgroup of *P*4/*nmm* (Wondratschek & Müller, 2004 ▸; Aroyo, 2016 ▸) [see Fig. 2 ▸(*b*)]. These two space groups are difficult to distinguish from each other using the systematic extinctions in PXRD. *P*4/*nmm* requires the reflection condition *hk*0: *h*+*k* = 2*n*, whereas *P* 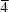
2_1_ *m* requires only *h*00: *h* = 2*n* and 0*k*0: *k* = 2*n* (Hahn, 2005 ▸). However, in the 2θ range up to 60°, the powder pattern contains only one reflection, which is systematically absent in *P*4/*nmm* but can be present in *P* 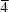
2_1_ *m*. This is the 210 reflection, which has an intensity of close to zero (see Fig. 1 ▸ *b*). Hence, an examination of the systematic extinctions left the space group ambiguous.

As a test for the space group, Rietveld refinements were performed in *P*4/*nmm* and *P* 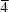
2_1_ *m* under identical conditions (identical treatment of background, profile parameters, anisotropic peak broadening, *etc*.). It is an interesting peculiarity that the number of structural parameters is identical in both space groups, which is a very rare case for an organic crystal structure in a group–subgroup relationship. Hence, the resulting confidence values of both space groups can be compared directly. The difference in the *R* values is slightly in favour of *P* 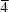
2_1_ *m* (see Table 1 ▸). The Rietveld plots are very similar, just the 111 reflection at 2θ = 30.28° is significantly better fitted in *P* 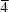
2_1_ *m* (see Fig. 1 ▸).

In both space groups, the structure is very similar, except for the disorder of the ethyl groups. In *P*4/*nmm* the ethyl group is disordered around a fourfold axis, which changes to a twofold axis in *P* 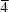
2_1_ *m* [see Figs. 2 ▸(*a*) and 2 ▸(*b*)].

As a further test for the space group, a Rietveld refinement was performed in the space group *P* 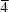
2_1_ *m* with two sets of ethyl groups, one in the position *x*, *x* + 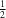, *z* (Wyckoff position 8*j*) according to the *P* 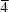
2_1_ *m* structure, and the other on the position −*x*, *x* + 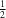, *z*, which is occupied in *P*4/*nmm*, but not in *P* 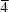
2_1_ *m* (see Fig. 2 ▸). The occupancies of both sets were set at *p* and 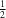 − *p*. For *P* 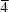
2_1_ *m*, *p* would be 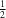 and for *P*4/*nmm*, *p* is 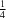. The parameter *p* refined to 0.476 (6). This value and the similarity of the *R* values between this refinement and the refinement in *P* 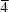
2_1_ *m* clearly indicate that, within the limitations of the powder data, the correct space group is *P* 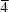
2_1_ *m* instead of *P*4/*nmm*.

**Figure 3:**
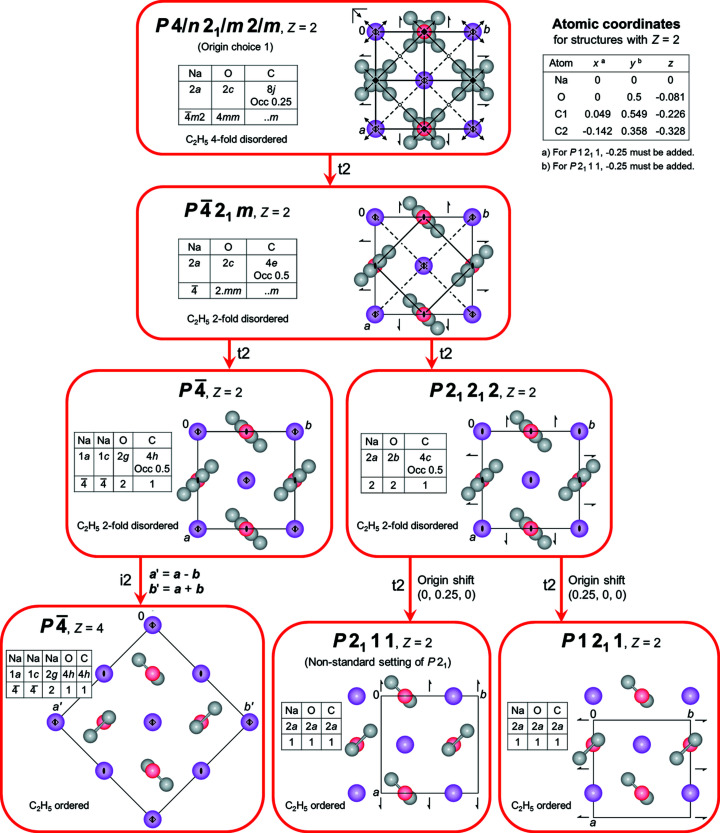
The Bärnighausen tree of NaOEt. Colour key: Na violet, O red and C grey. H atoms have been omitted for clarity. The view direction is [001]. t2 denotes a *translationengleiche* subgroup of index two and i2 an isomorphous subgroup of index two. The small tables give the atom types, Wyckoff positions and site symmetries. Occ denotes the occupancy, if different from one. The experimental crystal symmetry is *P* 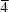
2_1_ *m*, with *Z* = 2.

A complete ordering of the ethyl groups would require further reduction of symmetry, *e.g.* to *P* 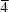 or *P*2_1_. The corresponding Bärnighausen tree (Bärnighausen, 1980 ▸; Chapuis, 1992 ▸; Müller, 2004 ▸, 2006 ▸, 2012 ▸) is shown in Fig. 3 ▸. Such a symmetry reduction would result in a deviation from tetragonal symmetry and/or in a larger unit cell (supercell). Both effects should be clearly visible in the powder pattern. However, the powder pattern of NaOEt gave no indication of either effect. Hence, the space group is likely to be *P* 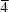
2_1_ *m*.

A transition into a *translationengleiche* subgoup of index 2 is frequently associated with twinning. Correspondingly, the NaOEt crystals may be twinned, *i.e.* in one domain the orientation of the ethyl groups is that shown in Fig. 2 ▸ *b* and in the other domain the groups are rotated by 90°. However, such a twinning cannot be observed by powder diffraction; hence, it remains unclear if the crystals are actually twinned.

**Figure 4:**
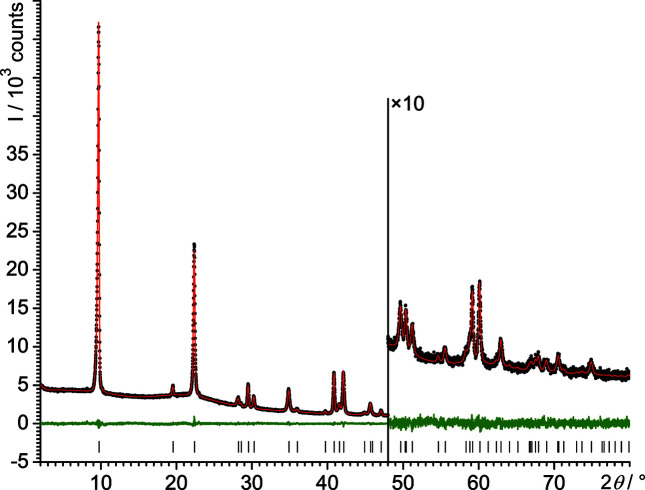
Final Rietveld plot of NaOEt. Experimental data are shown as black dots and simulated data as a red line, with the difference curve in green below. The vertical tick marks denote the reflection positions.

The final Rietveld refinements were carried out in *P* 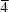
2_1_ *m*. No restraints were applied to the Na, C and O atoms. Restraints were only necessary for the H-atom positions. The final Rietveld plot is shown in Fig. 4 ▸. Crystallographic data are included in Table 2 ▸.

**Figure 5:**
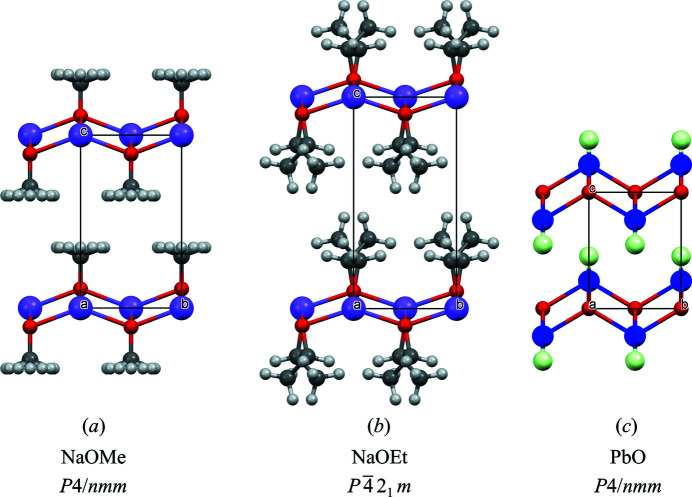
The crystal structures of (*a*) NaOMe, (*b*) NaOEt and (*c*) PbO (litharge). Colour key: Na violet, O red, C grey, H white and Pb blue. In part (*c*), the large light-green balls represent the lone pairs of the Pb^2+^ ions. The view direction is [100]. For a better comparison, the unit cell of NaOMe was shifted by (0, 0, 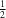) with respect to the original data of Weiss (1964 ▸) and the H atoms of NaOMe were added in calculated positions (with a fourfold disorder). Drawings were made with *Mercury* (Macrae *et al.*, 2020 ▸).

The crystal structure of NaOEt (Fig. 5 ▸ *b*) is similar to the structures of NaOMe and LiOMe (LiOMe type; Fig. 5 ▸ *a*). The Na^+^ ions form a quadratic net in the (001) plane. The O atoms are situated in the centre of each mesh 0.734 (3) Å above or below the plane. The ethyl groups point away from the nets on both sides; hence, they form covering nonpolar layers on both sides of the ionic Na–O nets. Subsequent layers are stacked in the [001] direction. This structure can be regarded as an anti-PbO structure. In red PbO (litharge; Boher *et al*., 1985 ▸), the Pb^2+^ and O^2−^ ions form the same net as the O and Na atoms in NaOEt, and the lone pairs of the Pb^2+^ ion in PbO resemble the positions of the ethyl groups in NaOEt (see Figs. 5*b* ▸ and 5*c* ▸).

LiOMe and NaOMe crystallize in the space group *P*4/*nmm* (*Z* = 2), with the methyl group on the fourfold axis, which causes no problems, because the shape of the methyl group is close to spherical. In contrast, in NaOEt, the crystal symmetry is reduced to *P* 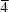
2_1_ *m*. Astonishingly, NaO*^n^*Pr and the higher sodium alkoxides again adopt the higher symmetry *P*4/*nmm* (*Z* = 2), with a fourfold disorder of the alkyl groups (see below). This raises the question, why does NaOEt not adopt *P*4/*nmm* symmetry? An ‘intuitive’ explanation for the lower symmetry of NaOEt would be that an ethyl group has a ‘less cylindrical’ shape than a methyl or propyl group and, hence, avoids being situated on a fourfold axis. The reduced disorder of the ethyl groups of NaOEt in *P* 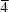
2_1_ *m* leads to a more efficient packing and a higher density. Actually, the density of NaOEt is 4% higher than the average density of NaOMe and NaO*^n^*Pr, both of which crystallize in *P*4/*nmm*.

### 3.2. Crystal structures, space group and disorder of NaO*^n^*Pr, NaO*^n^*Bu and NaO*^n^*Am   

Sodium *n*-propoxide (NaO*^n^*Pr), sodium *n*-butoxide (NaO*^n^*Bu) and sodium *n*-amylate (sodium *n*-pentoxide, NaO*^n^*Am) were synthesized from sodium and the corresponding alcohols. Upon evaporation of the alcoholic solutions, the solvates precipitated initially (as a mixture with the solvent-free phases). Further evaporation led to the solvent-free forms. The compounds are very sensitive to water; hence, any trace of moisture, also from air, had to be avoided during synthesis, evaporation and PXRD measurements.

The powder diagrams could be easily indexed with tetragonal unit cells, with *Z* = 2. The structures were solved by the real-space method and refined by the Rietveld method.

**Figure 6:**
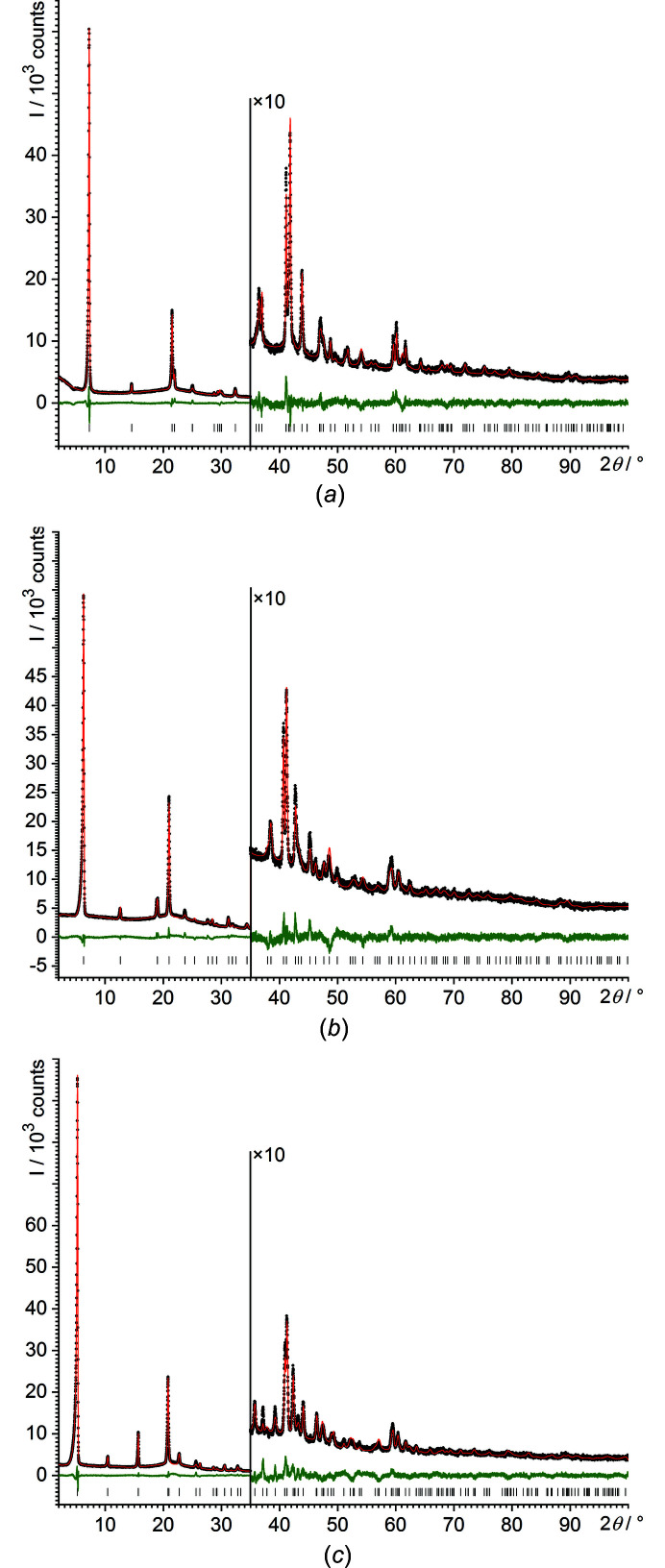
Final Rietveld plots of (*a*) NaO*^n^*Pr, (*b*) NaO*^n^*Bu and (*c*) NaO*^n^*Am. Experimental data are shown as black dots and simulated data as a red line, with the difference curve in green below. The vertical tick marks denote the reflection positions.

**Figure 7:**
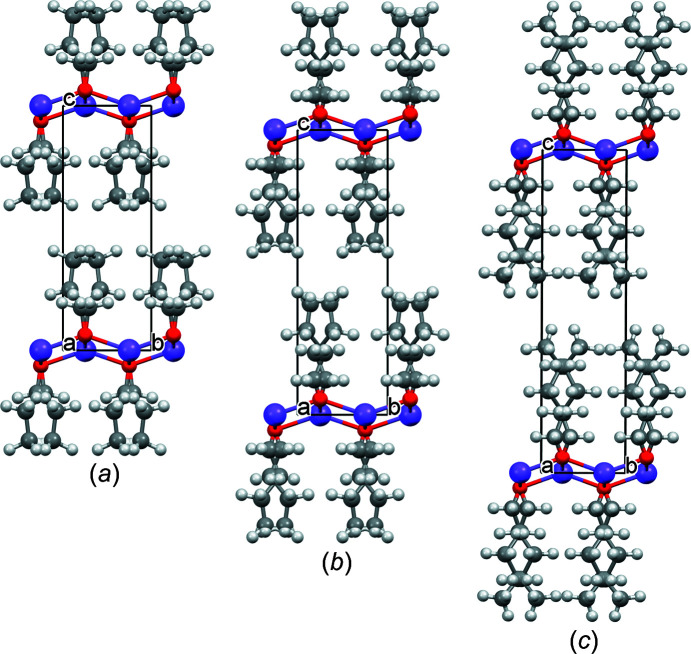
The crystal structures of (*a*) NaO*^n^*Pr, (*b*) NaO*^n^*Bu and (*c*) NaO*^n^*Am. Colour key: Na violet, O red, C grey and H white. The view direction is [100].

The crystal structures are similar to that of NaOEt (anti-PbO type). In the case of NaO*^n^*Pr, the space group is ambiguous. A refinement in *P* 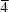
2_1_ *m* with two sets of alkyl groups, as performed for NaOEt, did not yield clear results. The occupancies refined to values of 0.38 (3) and 0.12 (3), which is exactly midway between 0.25 (for *P*4/*nmm*) and 0.5 (for *P* 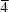
2_1_ *m*) (see Table 3 ▸). However, the refinement with one set of alkyl groups gave significantly lower confidence values in *P*4/*nmm* than in *P* 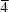
2_1_ *m*. For NaO*^n^*Bu and NaO*^n^*Am, refinements in *P*4/*nmm* also provided a better fit than in *P* 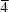
2_1_ *m* (Table 4 ▸). Correspondingly, the final refinements of all three compounds were performed in *P*4/*nmm*. The final Rietveld plots are shown in Fig. 6 ▸. Crystallographic data are included in Table 2 ▸. The crystal structures are shown in Fig. 7 ▸.

The lattice parameters and the space group of NaO*^n^*Pr agree with the data determined by Chandran *et al.* (2006 ▸).

The *n*-butyl and *n*-amyl groups are highly disordered, and the electron density is smeared out, especially for the terminal and the next-to-terminal C atoms. The description of these structures in *P*4/*nmm* with fourfold disordered alkyl groups on Wyckoff position 8*j* is only an approximation of the actual electron density.

We tried to prepare phase-pure powders of solvent-free NaO*^i^*Pr and NaO*^t^*Am, but the crystal structures could not be solved by PXRD yet. The samples probably contained mixtures of different phases.

### 3.3. Solvates   

By crystallization from the corresponding alcohols, we obtained single crystals of four alcohol solvates of sodium alkoxides: NaOEt·2EtOH, NaO*^n^*Pr·2*^n^*PrOH, NaO*^i^*Pr·5*^i^*PrOH and NaO*^t^*Am·*^t^*AmOH. All of these solvates are sensitive to moisture and air. In a vacuum and under argon (or nitro­gen), they decompose into their solvate-free forms. The decomposition is comparably slow for NaOEt·2EtOH, but fast for NaO*^i^*Pr·5*^i^*PrOH. Correspondingly, the solvates must be stored in their mother liquor or in the presence of vapours of the corresponding alcohols or kept at low temperature.

The chemical compositions and crystal structures of these four solvates were determined by single-crystal X-ray diffraction. However, there were three obstacles: (i) the mounting of the crystals on the diffractometer was challenging due to their sensitivity to air, moisture, vacuum and dry inert gas; (ii) the crystal quality was limited, especially for NaO*^i^*Pr·5*^i^*PrOH; (iii) the crystal structures of NaO*^n^*Pr·2*^n^*PrOH and NaO*^i^*Pr·5*^i^*PrOH are highly disordered. In NaO*^n^*Pr·2*^n^*PrOH, the disorder affects all of the propyl groups. In NaO*^i^*Pr·5*^i^*PrOH, four of the ten *^i^*PrOH units are disordered over two widely separated positions each.

The stability of the solvates of the sodium *n*-alkoxides decreases with increasing chain length. Correspondingly, crystal structures could be determined only for the solvates of NaOEt and NaO*^n^*Pr, whereas the solvates of NaO*^n^*Bu and NaO*^n^*Am are highly instable, poorly crystalline and decompose rapidly, even under cold dry nitro­gen.

#### 3.3.1. NaOEt·2EtOH and NaO*^n^*Pr·2*^n^*PrOH   

**Figure 8:**
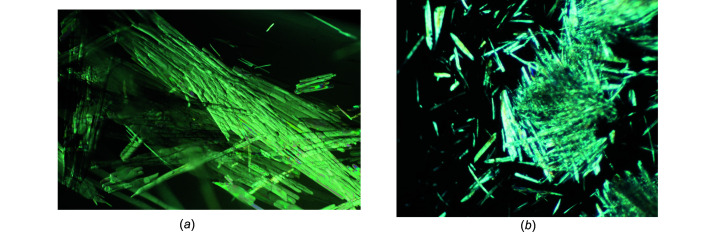
The crystals of (*a*) NaO*^n^*Pr·2*^n^*PrOH (image width about 15 mm) and (*b*) NaO*^t^*Am·*^t^*AmOH (image width about 2 mm), both in polarized light.

**Figure 9:**
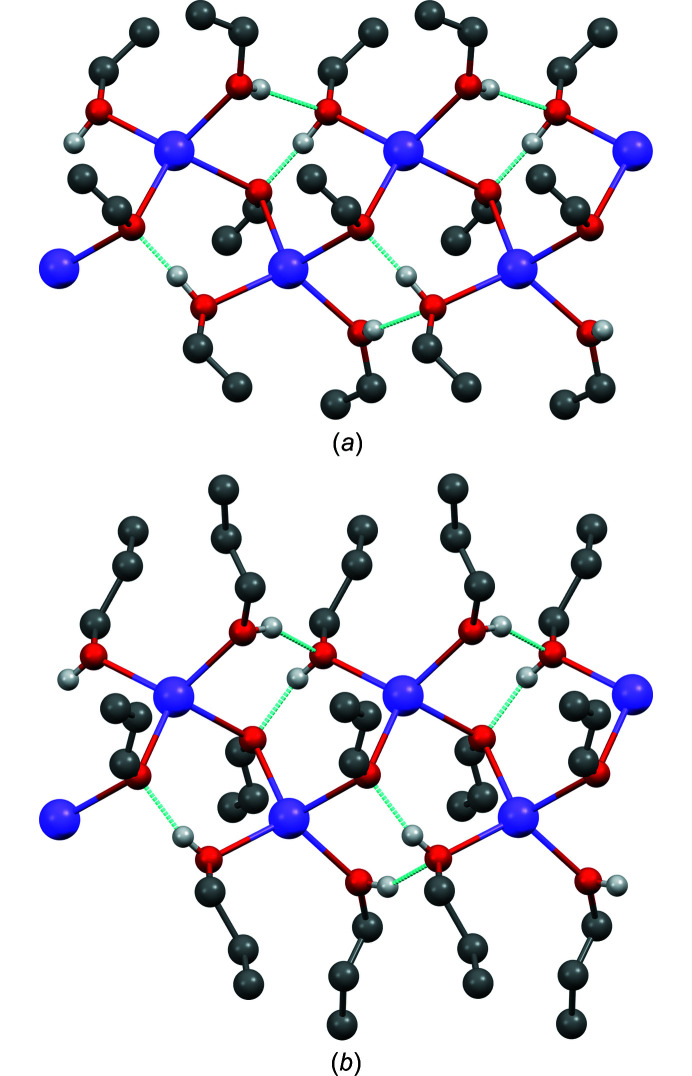
Helical chains in (*a*) NaOEt·2EtOH and (*b*) NaO*^n^*Pr·2*^n^*PrOH. Colour key: Na violet, O red, C grey and H white. Hydrogen bonds are drawn as dotted light-blue lines. The H atoms of the alkyl groups have been omitted for clarity. In part (*b*), the disordered propyl groups are represented by their major-occupied atomic positions only.

**Figure 10:**
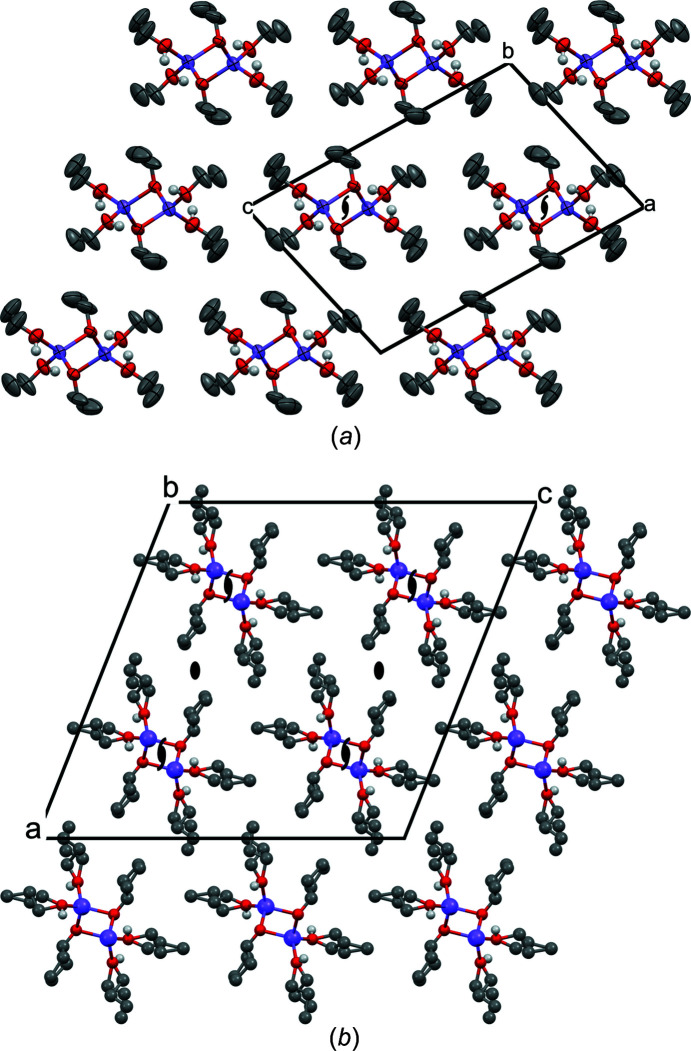
The crystal structures of (*a*) NaOEt·2EtOH (space group *P*2_1_/*c*, view direction [010] and displacement ellipsoids at the 50% probability level) and (*b*) NaO*^n^*Pr·2*^n^*PrOH (space group *C*2/*c*, view direction [0
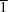
0]). Selected symmetry elements are shown. In both structures, there is a 2_1_ screw axis in the middle of each chain.

Sodium ethox­ide and sodium *n*-propoxide crystallize as needles (see Fig. 8 ▸). The single-crystal X-ray analysis (Table 5 ▸) revealed the com­pounds to be disolvates with the composition NaO*R*·2*R*OH, as determined by Geuther (1868*a* ▸,*b* ▸) and Frölich (Geuther & Frölich, 1880 ▸). Both solvates are isostructural. The O atom of the alkoxide anion (*R*O^−^) bridges two Na^+^ ions, leading to helical Na—O—Na—O chains. The chains follow a crystallographic 2_1_ screw axis. The Na^+^ ions are additionally coordinated to two alcohol molecules (*R*OH), resulting in a distorted tetrahedral coordination geometry for the Na^+^ ions. In NaO*^n^*Pr·2*^n^*PrOH, all the propyl groups are disordered and most of the C atoms were refined with split positions, with occupancies between 0.40 and 0.60. The OH groups of the alcohol molecules form hydrogen bonds to neighbouring *R*OH molecules and *R*O^−^ anions, which additionally stabilize the chains (see Fig. 9 ▸). The alkyl groups point outwards. Hence the chains are like tubes, with a polar/ionic inner region and a nonpolar outer region. In the crystal, all the tubes are arranged parallel and form a distorted hexagonal packing (see Fig. 10 ▸). However, the space group is different, *i.e.* *P*2_1_/*c* for NaOEt·2EtOH and *C*2/*c* for NaO*^n^*Pr·2*^n^*PrOH.

Between the ‘tubes’ there are only van der Waals contacts between the alkyl groups. This structure explains the observed needle-like morphology of both compounds, with the needle axes parallel to the chain direction [010]. The weak interactions between the ‘tubes’ allow them to librate around their long axis, which is manifested in the anisotropic displacement parameters of NaOEt·2EtOH (see Fig. 10 ▸ *a*). In the case of NaO*^n^*Pr·2*^n^*PrOH, the limited crystal quality and the disorder of the propyl groups prevent an interpretation of the displacement ellipsoids.

The corresponding lithium methoxide solvate, LiOMe·2MeOH, is a disolvate, like NaOEt·2EtOH and NaO*^n^*Pr·2*^n^*PrOH, but its structure is different. LiOMe·2MeOH consists of Li_4_(OMe)_4_(MeOH)_6_ tetramers, which are connected through hydrogen bonds *via* MeOH molecules to form a two-dimensional network. As in NaOEt, the interior layer of this network consists of metal ions and O atoms, whereas the alkyl groups point outwards. These layers are stacked through van der Waals contacts between the methyl groups only.

#### 3.3.2. NaO*^i^*Pr·5*^i^*PrOH   

Sodium isopropoxide forms a solvate which contains as many as five molecules of iso­propanol per NaO*^i^*Pr unit. Hence, this structure can be regarded as an iso­propanol in which one sixth of the protons of the OH groups are replaced by sodium ions. Correspondingly, the structure of NaO*^i^*Pr·5*^i^*PrOH provides an insight into the structure of liquid iso­propanol itself.

**Figure 11:**
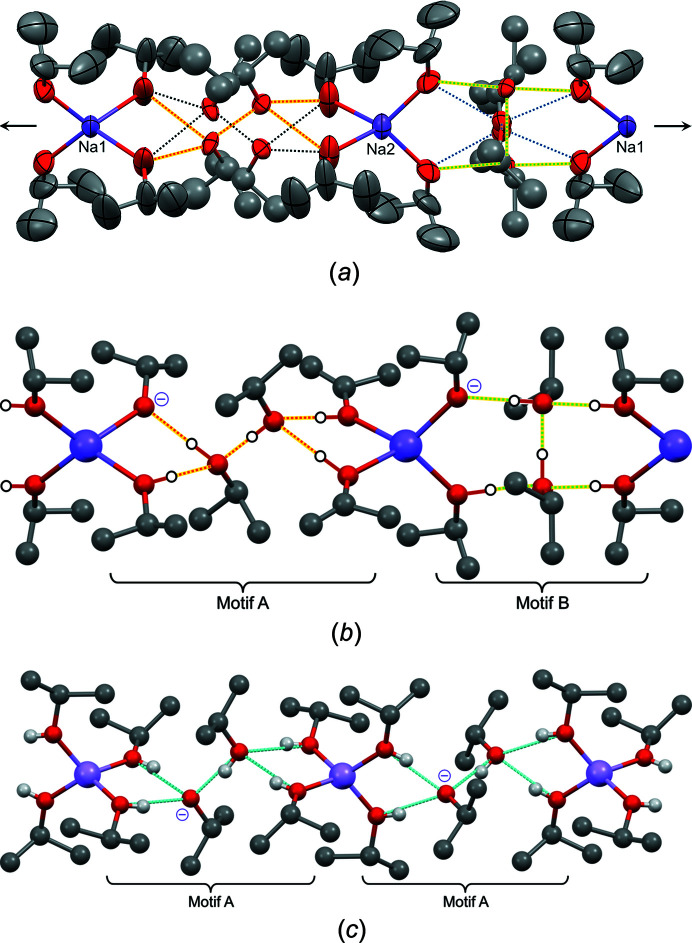
(*a*) The crystal structure of NaO*^i^*Pr·5*^i^*PrOH, showing one chain. The view direction is [001], with the *b* axis horizontal. Displacement ellipsoids are drawn at the 20% probability level. All *^i^*PrOH molecules not directly coordinated to Na have an occupancy of 0.5 only. Dotted lines represent the four independent hydrogen-bond networks. The arrows indicate the crystallographic twofold axis. (*b*) Selected hydrogen-bond networks in NaO*^i^*Pr·5*^i^*PrOH. (*c*) The crystal structure of LiO*^i^*Pr·5*^i^*PrOH. The disorder of the *^i^*Pr groups is not shown. In parts (*b*) and (*c*), the H atoms are shown as white spheres in calculated positions. The H-atom positions shown here represent only one possibility; actually, the H atoms are disordered and could not be located experimentally. Minus (−) signs denote the anions.

The solvate crystallizes in the space group *C*2/*c*, with *Z* = 8. There are two symmetrically independent Na^+^ ions, both on the twofold axis. Each Na^+^ ion coordinates to four O atoms of *^i^*PrO^−^ and *^i^*PrOH moieties (Fig. 11 ▸ *a*). The H atoms of the OH groups could not be located reliably, as they are probably dynamically disordered. Because of the charge compensation, it is expected that in any instance each Na^+^ cation is surrounded by one *^i^*PrO^−^ ligand and three *^i^*PrOH molecules. These Na(*^i^*PrO)(*^i^*PrOH)_3_ units are connected by further *^i^*PrOH molecules to form a chain along the twofold axis parallel to [010] (see Figs. 11 ▸ *a* and 11 ▸ *b*). All iso­propanol molecules which are not directly coordinated to sodium have an occupancy of 0.5. All iso­propyl groups show disorder.

The geometry of the chain is close to 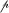
112/*m* symmetry (rod group No. 11; Kopský & Litvin, 2010 ▸).

There are four independent hydrogen-bond networks in each chain, each with an occupancy of 0.5 (see Fig. 11 ▸ *a*). Two of these networks are shown in Fig. 11 ▸ *b*.

**Figure 12:**
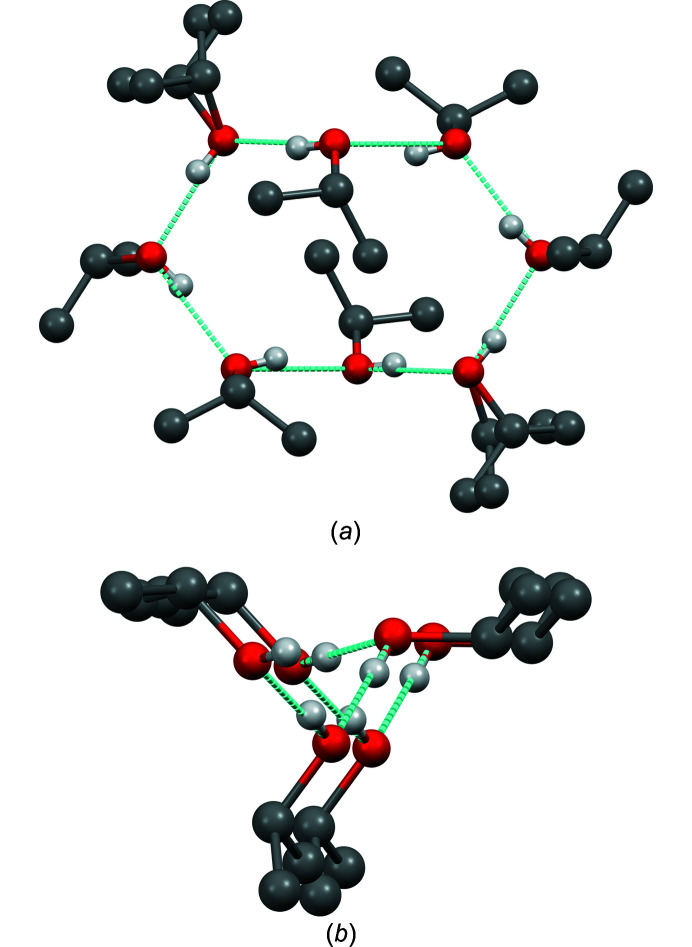
The hydrogen-bond networks of solid pure iso­propanol. (*a*) Eight-membered ring in the high-pressure phase. (*b*) Threefold screw axis in the low-temperature phase. In both structures, some of the iso­propyl groups are disordered.

The hydrogen-bond networks are considerably different from those in pure solid iso­propanol. Pure iso­propanol forms different hydrogen-bond networks, depending on the experimental conditions: the high-pressure polymorph exhibits eight-membered rings, whereas the low-temperature polymorph forms helical chains with local 3_1_ or 3_2_ symmetry (see Fig. 12 ▸) (Ridout & Probert, 2014 ▸). In contrast, the hydrogen-bond network of NaO*^i^*Pr·5*^i^*PrOH contains branching between the alcohol molecules, *i.e.* alcohol molecules are connected by hydrogen bonds to three other alcohol molecules. There are two different topologies, marked as ‘Motif A’ and ‘Motif B’ in Fig. 11 ▸(*b*). Motif A is also present in LiO*^i^*Pr·5*^i^*PrOH (Mehring *et al.*, 2002 ▸). Motifs A and B can neither be found in other sodium alkoxide solvates nor in the crystal structures of pure iso­propanol. In liquid iso­propanol, one could expect to find a mixture of all three motifs, namely, rings, chains and branchings.

**Figure 13:**
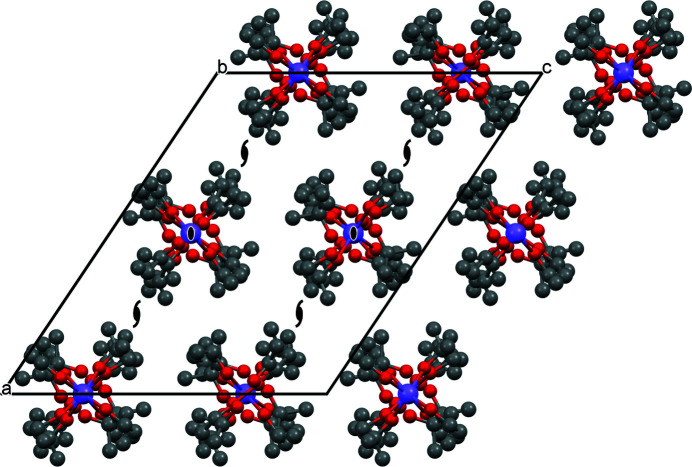
The crystal structure of NaO*^i^*Pr·5*^i^*PrOH (space group *C*2/*c*, view along the chains and view direction [0
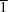
0]). The chains are located on twofold rotation axes, in contrast to the 2_1_ screw axes for NaO*^n^*Pr·2*^n^*PrOH (see Fig. 10 ▸ *b*).

In NaO*^i^*Pr·5*^i^*PrOH, the hydrogen-bonded chains are sur­rounded by the nonpolar iso­propyl groups. Between the chains, there are only van der Waals contacts between the iso­propyl groups (see Fig. 13 ▸). Similarly, in both polymorphs of pure *^i^*PrOH, the rings and chains have polar surfaces, and are connected to neighbouring rings or chains by van der Waals interactions (see Figs. S1 and S2 in the supporting information).

The chains of NaO*^i^*Pr·5*^i^*PrOH are arranged in a distorted hexagonal packing. The packing seems to be similar to the chain packing in NaO*^n^*Pr·2*^n^*PrOH. The space group is also the same (*i.e.* *C*2/*c*). However, the chains in NaO*^i^*Pr·5*^i^*PrOH are situated on a twofold rotation axis, whereas the chains of NaO*^n^*Pr·2*^n^*PrOH are placed on a 2_1_ screw axis (see Figs. 10 ▸ *b* and 13 ▸).

#### 3.3.3. NaO*^t^*Am·*^t^*AmOH   

**Figure 14:**
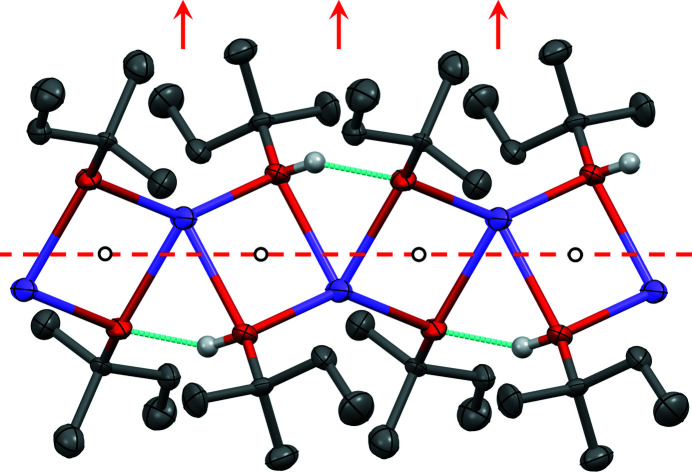
A view of the structure of NaO*^t^*Am·*^t^*AmOH, with displacement ellipsoids drawn at the 50% probability level. The H atoms of the *tert*-amyl groups have been omitted for clarity. The light-blue lines denote the hydrogen bonds. The H atom is disordered along the hydrogen bond. The black circles represent crystallographic inversion centres. The red symmetry elements represent the approximated local symmetry of the chain (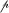
2/*b*11).

The X-ray structure analysis revealed that sodium *tert*-amylate (sodium 2-methyl-2-butano­late, NaO*^t^*Am) forms a monosolvate with *tert*-amyl alcohol (2-methyl-2-butanol). In this structure, neighbouring Na^+^ ions are bridged by two ligands in the form of a square. The square shares corners with two other squares, leading to chains (see Fig. 14 ▸). Each square is centred by a crystallographic inversion centre. The squares are additionally connected by hydrogen bonds. The H atom engaged in this bond is probably disordered, so that in any instance each square contains one *^t^*AmO^−^ anion and one *^t^*AmOH ligand, due to electrostatic considerations. The squares form an interplanar angle of 49.8° only, which is apparently caused by the hydrogen bonds. The *tert*-amyl groups point outwards, as in the other solvates.

The chain has approximately 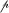
2/*b*11 symmetry, which is a nonstandard setting of 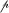
2/*c*11 (rod group No. 7; Kopský & Litvin, 2010 ▸). In the crystal, only the inversion symmetry is maintained in the space-group symmetry *P*12_1_/*n*1.

**Figure 15:**
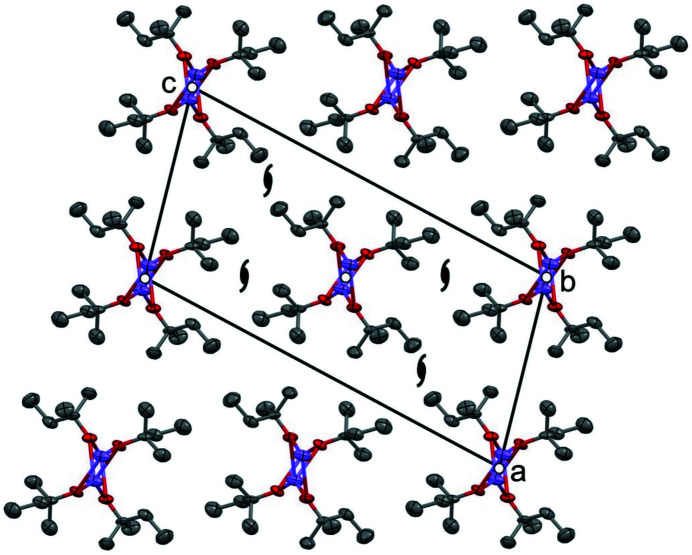
The crystal structure of NaO*^t^*Am·*^t^*AmOH, viewed along the chains (space group *P*2_1_/*n*, view direction [010]), with displacement ellipsoids drawn at the 50% probability level. Selected symmetry elements are shown.

The arrangement of the chains (Fig. 15 ▸) is similar to that in NaOEt·2EtOH (Fig. 10 ▸ *a*). The space group is also the same (*P*2_1_/*c*, here in the *P*2_1_/*n* setting). However, in NaOEt·2EtOH, the chains are aligned along the 2_1_ axis, whereas in NaO*^t^*Am·*^t^*AmOH, the chains contain inversion centres.

## 4. Conclusion   

In this study, we determined the crystal structures of a series of sodium alkoxides NaO*R* (*R* = Et, *^n^*Pr, *^n^*Bu and *^n^*Am) and of a series of solvates of the composition NaO*R*·*x* *R*OH (*R* = Et, *^n^*Pr, *^i^*Pr and *^t^*Am; *x* = 1, 2 and 5). Surprisingly, the crystal structures were unknown. Only the structures of NaOMe, NaO*^t^*Bu and NaOMe·2MeOH had been determined previously.

The solvates show a variety of compositions, from the monosolvate NaO*^t^*Am·*^t^*AmOH *via* the disolvates NaO*R*·2*R*OH (*R* = Et and *^n^*Pr) to the pentasolvate NaO*^i^*Pr·5*^i^*PrOH. The solvates were obtained from saturated solutions of the alkoxides in the corresponding alcohols. We did not systematically investigate a variation of compositions, temperature and vapour pressure. Presumably, other solvate phases with different compositions may also exist.

In all the solvated and solvate-free structures, the Na^+^ ion is coordinated in a distorted tetrahedral geometry to four O atoms. In the solvent-free compounds NaO*R*, the O atom has five neighbouring atoms: four Na^+^ ions and the alkyl group. Such a fivefold coordination is quite unusual for organic O atoms. The O atoms in organic compounds generally have a maximum of three or four neighbours, when counting counter-ions, alkyl groups, H atoms and accepted hydrogen bonds. In most crystal structures of alcohols *R*OH, the O atoms have only three neighbours: one alkyl group, one H atom and one hydrogen bond as acceptor. The overcrowded coordination of the O atoms in sodium alkoxides is the reason why they so readily form solvates. Already with one additional alcohol molecule, the coordination number of oxygen drops from 5 to 4, as can be seen in NaO*^t^*Am·*^t^*AmOH. Any additional alcohol molecule increases the number of threefold-coordinated O atoms. This stabilization is reflected in the thermal stability of the solvates: to obtain the solvent-free alkoxides, NaOEt·2EtOH must be heated at ambient pressure to about 200 °C and NaO*^n^*Pr·*^n^*PrOH even to about 220 °C (Geuther & Frölich, 1880 ▸). In contrast to sodium alkoxides, the sodium alkanoates *R*COO^−^·Na^+^ and sulfonates *R*SO_3_^−^·Na^+^ rarely form solvates with alcohol or *R*COOH molecules. There, the higher number of O atoms provides a sufficient number of donor atoms for the coordination of the Na^+^ ion, even with higher coordination numbers of Na^+^, *e.g.* 6 in CH_3_COO^−^·Na^+^ (Dittrich *et al.*, 2018 ▸) or 6–7 in CH_3_SO_3_^−^·Na^+^ (Wei & Hingerty, 1981 ▸).

Four different topologies are present in sodium alkoxides: the alkoxides with linear alkyl chains (*R* = Me, Et, *^n^*Pr, *^n^*Bu and *^n^*Am) form layers of Na^+^ and O^−^ atoms, NaO*^t^*Bu forms clusters (hexamers and nonamers), NaOMe·2MeOH forms tetramers, which are connected by hydrogen bonds into layers, and the other solvates form chains of differing composition. In all cases, polar and nonpolar regions are clearly separated: the interior of the layers, clusters and chains consist of Na^+^ and –O^−^ ions and is held together by electrostatic forces, whereas the outer regions are composed of the nonpolar alkyl groups. Neighbouring layers, clusters or chains are connected by van der Waals contacts only. As a result, the chain structures of all the solvates form needles. NaO*^t^*Bu forms prisms or cubes. For the layer structures of NaO*R*, a plate-like morphology could be expected.

The compound NaO*^i^*Pr·5*^i^*PrOH differs from pure isobutanol only by the substitution of every sixth proton of an OH group with a sodium cation. The crystal structure exhibits a complicated chain structure with branched hydrogen bonds between the iso­propanol molecules. Such branchings are not present in the crystal structures of pure iso­propanol, but give an interesting insight into the structural diversity of liquid iso­propanol.

## Supplementary Material

Crystal structure: contains datablock(s) global, NaOEt, NaOEt2EtOH, NaOnPr, NaOnBu, NaOnAm, NaOiPr_5HOiPr, NaOnPr_2HOnPr, NaOtAm_HOtAm. DOI: 10.1107/S205252062001584X/ne5003sup1.cif

Structure factors: contains datablock(s) NaOEt. DOI: 10.1107/S205252062001584X/ne5003NaOEtsup2.hkl

Structure factors: contains datablock(s) NaOEt2EtOH. DOI: 10.1107/S205252062001584X/ne5003NaOEt2EtOHsup3.hkl

Structure factors: contains datablock(s) NaOnPr. DOI: 10.1107/S205252062001584X/ne5003NaOnPrsup4.hkl

Structure factors: contains datablock(s) NaOnBu. DOI: 10.1107/S205252062001584X/ne5003NaOnBusup5.hkl

Structure factors: contains datablock(s) NaOnAm. DOI: 10.1107/S205252062001584X/ne5003NaOnAmsup6.hkl

Structure factors: contains datablock(s) NaOiPr_5HOiPr. DOI: 10.1107/S205252062001584X/ne5003NaOiPr_5HOiPrsup7.hkl

Structure factors: contains datablock(s) NaOnPr_2HOnPr. DOI: 10.1107/S205252062001584X/ne5003NaOnPr_2HOnPrsup8.hkl

Structure factors: contains datablock(s) NaOtAm_HOtAm. DOI: 10.1107/S205252062001584X/ne5003NaOtAm_HOtAmsup9.hkl

Supporting information file. DOI: 10.1107/S205252062001584X/ne5003NaOEtsup10.cml

Supporting information file. DOI: 10.1107/S205252062001584X/ne5003NaOEt2EtOHsup11.cml

Supporting information file. DOI: 10.1107/S205252062001584X/ne5003sup12.pdf

CCDC references: 1943793, 1943794, 1998221, 1998220, 1998219, 1998224, 1998225, 1998227

## Figures and Tables

**Table 1:** Rietveld refinement of NaOEt in *P*4/*nmm* and *P* 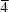
2_1_ *m* under identical conditions, with restrained H-atom positions and a 2θ range of 2–60° The values marked by a ′ are background-subtracted values. *N*(param) is the number of structural parameters, including the occupancy parameter.

	*P*4/*nmm*	*P* 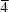 2_1_ *m*	*P* 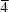 2_1_ *m* with both orientations of Et
*R*_wp_ (%)	4.320	4.146	4.149
*R*_wp_′ (%)	16.98	16.30	16.30
*R*_p_ (%)	3.205	3.122	3.126
*R*_p_′ (%)	18.21	17.74	17.75
Goodness-of-fit	2.016	1.935	1.936
*N*(param)	13	13	13 + 1 (occupancy)
Occupancy of the ethyl group	0.25 (fixed)	0.5 (fixed)	0.476 (6):0.024 (6)

**Table 2:** Experimental details for NaOMe, NaOEt, NaO*^n^*Pr, NaO*^n^*Bu and NaO*^n^*Am

	**NaOMe** (Weiss, 1964 ▸)	**NaOEt**	**NaO*^n^*Pr**	**NaO*^n^*Bu**	**NaO*^n^*Am**
Crystal data					
Chemical formula	CH_3_ONa	C_2_H_5_ONa	C_3_H_7_ONa	C_4_H_9_ONa	C_5_H_11_ONa
CCDC number	–	1943793	1998221	1998220	1998219
*M*_r_	54.02	68.05	82.08	96.10	109.12
Crystal system	Tetragonal	Tetragonal	Tetragonal	Tetragonal	Tetragonal
Space group (No.)	*P*4/*nmm* (129)	*P* 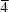 2_1_ *m* (113)	*P*4/*nmm* (129)	*P*4/*nmm* (129)	*P*4/*nmm* (129)
*Z*, *Z*′	2, 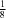	2, 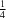	2, 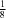	2, 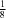	2, 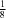
Temperature (K)	298	298	298	298	298
*a* (Å)	4.343 (5)	4.41084 (4)	4. 38439 (5)	4.43232 (9)	4.4084 (2)
*c* (Å)	7.432 (10)	9.06779 (17)	12.1431 (3)	14.0143 (9)	16.9376 (12)
*V* (Å^3^)	140.2 (2)	176.418 (5)	233.426 (8)	275.318 (19)	329.16 (4)
ρ_calc_ (10^3^ kg m^−3^)	1.28	1.28	1.17	1.16	1.11
Radiation type	Cu *Kα*	Cu *Kα*_1_	Cu *Kα*_1_	Cu *Kα*_1_	Cu *Kα*_1_
Wavelength (Å)	1.5418	1.5406	1.5406	1.5406	1.5406
μ (mm^−1^)	–	1.845	1.473	1.314	1.154
					
Data collection					
Diffractometer	Goniometer with counting tube	Stoe Stadi-P	Stoe Stadi-P	Stoe Stadi-P	Stoe Stadi-P
Specimen mounting	Powder in N_2_ stream between polymer films	1.0 mm glass capillary	1.0 mm glass capillary	1.0 mm glass capillary	1.0 mm glass capillary
Data collection mode	Transmission	Transmission	Transmission	Transmission	Transmission
Detector	Counting tube	Linear position-sensitive	Linear position-sensitive	Linear position-sensitive	Linear position-sensitive
2θ_min_ (°)	11	2.0	2.0	2.0	2.0
2θ_max_ (°)	103	80.0	100	100	100
2θ_step_ (°)	–	0.01	0.01	0.01	0.01
					
Refinement					
*R*_p_	0.159	0.0233	0.0373	0.0347	0.0376
*R*_wp_	–	0.0339	0.0479	0.0471	0.0535
*R*_exp_	–	0.0214	0.0295	0.0240	0.0273
*R*_p_′^(*a*)^	–	0.133	0.161	0.175	0.120
*R*_wp_′^(*a*)^	–	0.134	0.159	0.187	0.158
*R*_exp_′^(*a*)^	*–*	0.085	0.0981	0.0951	0.0806
Goodness-of-fit	*–*	1.42	1.62	1.96	1.96
No. of data points	64 observed intensities	7800	9800	9800	9800
No. of parameters	4	40	56	61	72
No. of restraints	0	8	11	21	26
H-atom treatment	*f*_H_ included in C atom	Refined with restraints	Refined with restraints	Refined with restraints	Refined with restraints

Note: (*a*) *R*_p_′, *R*_wp_′ and *R*_exp_′ values are background-corrected data according to Coelho (2018).

**Table 3:** Rietveld refinement of NaO*^n^*Pr in *P*4/*nmm* and *P* 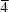
2_1_ *m* under identical conditions, with restraints on the C and H atoms The values marked by a ′ are background-subtracted values. The last column denotes a refinement in *P* 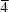
2_1_ *m* with two sets of propyl groups, one corresponding to the orientation in *P* 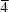
2_1_ *m* and the other rotated by 90°, which is occupied in *P*4/*nmm*, but empty in *P* 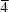
2_1_ *m*. *N*(param) is the number of structural parameters, including the occupancy parameter.

	*P*4/*nmm*	*P* 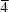 2_1_ *m*	*P* 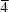 2_1_ *m* with both orientations of ^*n*^Pr
*R*_wp_ (%)	5.424	5.654	5.386
*R*_wp_′ (%)	17.953	18.714	17.832
*R*_p_ (%)	4.198	4.376	4.238
*R*_p_′ (%)	18.139	18.908	18.324
Goodness-of-fit	1.837	1.915	1.824
*N*(param)	18	18	18 + 1 (occupancy)
Occupancy *p*	0.25 (fix)	0.5 (fix)	0.38 (3):0.12 (3)

**Table 4:** Rietveld refinement of NaO*^n^*Bu and NaO*^n^*Am in *P*4/*nmm* and *P* 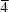
2_1_ *m* under identical conditions, with restraints on the C and H atoms *N*(param) is the number of structural parameters, including the occupancy parameter.

	NaO*^n^*Bu		NaO*^n^*Am	
	*P*4/*nmm*	*P* 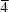 2_1_ *m*	*P*4/*nmm*	*P* 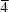 2_1_ *m*
*R*_wp_ (%)	4.620	4.884	5.403	5.762
*R*_wp_′ (%)	18.52	19.30	15.87	17.03
*R*_p_ (%)	3.578	3.766	3.875	4.071
*R*_p_′ (%)	18.73	19.11	12.44	13.13
Goodness-of-fit [*S*]	1.924	2.032	1.974	2.106
*N*(param)	23	23	28	28

**Table 5:** Experimental details for the sodium alkoxide solvates All determinations were carried out with Cu *K*α radiation using a Siemens Bruker three-circle diffractometer with an APEXII detector, an Incoatec Iμs microfocus source and mirror optics.

	**NaOEt·2EtOH**	**NaO*^n^*Pr·2*^n^*PrOH**	**NaO*^i^*Pr·5*^i^*PrOH**	**NaO*^t^*Am·*^t^*AmOH**
Crystal data				
Chemical formula	C_2_H_5_ONa·2C_2_H_5_OH	C_3_H_7_ONa·2C_3_H_7_OH	C_3_H_7_ONa·5C_3_H_7_OH	C_5_H_11_ONa·C_5_H_11_OH
CCDC number	1943794	1998225	1998224	1998227
*M*_r_	160.18	202.26	382.55	198.27
Crystal system	Monoclinic	Monoclinic	Monoclinic	Monoclinic
Space group (No.)	*P*2_1_/*n* (14)	*C*2/*c* (15)	*C*2/*c* (15)	*P*2_1_/*c* (14)
*Z*, *Z*′	4, 1	8, 1	8, 1	4, 1
Temperature (K)	238 (2)	173 (2)	173 (2)	296 (2)
*a* (Å)	11.622 (6)	23.745 (5)	21.2073 (18)	10.1260 (8)
*b* (Å)	5.1926 (9)	5.0750 (11)	17.1307 (13)	6.0299 (5)
*c* (Å)	17.682 (6)	24.174 (5)	17.825 (2)	20.6944 (18)
β (°)	104.08 (3)	111.589 (10)	123.871 (5)	104.16
*V* (Å^3^)	1035.0 (7)	2708.7 (10)	5376.9 (10)	1225.18 (18)
ρ_calc_ (10^3^ kg m^−3^)	1.03	0.992	0.945	1.08
Wavelength (Å)	1.54178	1.54178	1.54178	1.54178
μ (mm^−1^)	1.006	0.849	0.686	0.869
Crystal habit	Needle	Needle	Needle	Needle
Crystal size (mm)	0.8 × 0.08 × 0.02	0.5 × 0.05 × 0.05	0.4 × 0.02 × 0.02	1 × 0.02 × 0.01
				
Data collection				
θ range (°)	4.14–59.9	3.93–50.9	3.60–40.2	4.41–50.7
Absorption correction	Multi-scan (*SADABS*; Bruker, 2015 ▸)			
*T*_min_, *T*_max_	0.2700, 0.7486	0.5182, 0.7500	0.771, 0.875	0.477, 0.991
No. of measured reflections	5735	10 777	9647	13194
No. of unique reflections	1124	1616	1652	1300
*R*_int_	0.317	0.0887	0.0724	0.112
				
Refinement				
No. of parameters	95	114	239	118
No. of restraints	0	0	84	15
*wR*(*F* ^2^)	0.2709	0.2984	0.3669	0.1613
*R*[*F* ^2^ > 2σ(*F* ^2^)]	0.2178	0.0954	0.1215	0.0574
*S*	0.980	1.064	1.196	1.002
Δρ_max_, Δρ_min_ (e^−^ Å^−3^)	0.16, −0.20	0.24, −0.20	0.22, −0.17	0.30, −0.26

## Footnotes

1 For a discussion on the name ‘Rietveld method’ *versus* the name ‘Loopstra method’, see van Laar & Schenk (2018 ▸).

## References

[bb1] Aroyo, M. I. (2016). *International Tables for Crystallography*, Vol. A, *Space-group Symmetry*, 6th ed. Chester: International Union of Crystallography.

[bb2] Bärnighausen, H. (1980). *MATCH Commun. Math. Comput. Chem.* **9**, 139–175.

[bb44] Beske, M., Tapmeyer, L. & Schmidt, M. U. (2020). *Chem. Commun.* **56**, 3520–3523. 10.1039/c9cc08907a 32101200

[bb3] Blanchard, J.-M., Bousquet, J., Claudy, P. & Letoffe, J.-M. (1976). *J. Therm. Anal.* **9**, 191–203.

[bb4] Boher, P., Garnier, P., Gavarri, J. R. & Hewat, A. W. (1985). *J. Solid State Chem.* **57**, 343–350.

[bb5] Boultif, A. & Louër, D. (1991). *J. Appl. Cryst.* **24**, 987–993.

[bb6] Bruker (2015). *APEX3*. Bruker AXS GmbH, Karlsruhe, Germany.

[bb8] Chandran, K., Nithya, R., Sankaran, K., Gopalan, A. & Ganesan, V. (2006). *Bull. Mater. Sci.* **29**, 173–179.

[bb9] Chapuis, G. C. (1992). *Symmetry relationships between crystal structures and their practical application*, in *Modern Perspectives in Inorganic Chemistry*, edited by E. Parté, pp. 1–16. Dordrecht: Kluwer Academic Publishers.

[bb11] Coelho, A. A. (2018). *J. Appl. Cryst.* **51**, 210–218.

[bb12] David, W. I. F., Shankland, K., van de Streek, J., Pidcock, E., Motherwell, W. D. S. & Cole, J. C. (2006). *J. Appl. Cryst.* **39**, 910–915.

[bb13] Davies, J. E., Kopf, J. & Weiss, E. (1982). *Acta Cryst.* B**38**, 2251–2253.

[bb14] Dittrich, B., Bergmann, J., Roloff, P. & Reiss, G. J. (2018). *Crystals*, **8**, 213–224.

[bb15] Friedrich, H., Guth, J., Schweinzer, J., Letzelter, T. & Bender, H.-J. (1999). European Patent EP 1086067 B1.

[bb16] Geuther, A. (1859). *Justus Liebigs Ann. Chem.* **109**, 71–79.

[bb17] Geuther, A. (1868*a*). *Jena. Z. Med. Naturwiss.* **4**, 16–18.

[bb18] Geuther, A. (1868*b*). *Jena. Z. Med. Naturwiss.* **4**, 241–263.

[bb19] Geuther, A. & Frölich, O. (1880). *Justus Liebigs Ann. Chem.* **202**, 288–331.

[bb20] Greiser, T. & Weiss, E. (1977). *Chem. Ber.* **110**, 3388–3396.

[bb21] Hahn, T. (2005). Editor. *International Tables for Crystallography*, Vol. A, *Space-group symmetry*, 5th ed., with corrections. Chester: International Union of Crystallography.

[bb22] Hofmann, D. W. M. (2002). *Acta Cryst.* B**58**, 489–493. 10.1107/s0108768101021814 12037338

[bb23] Hunger, K. & Schmidt, M. U. (2018). In *Industrial Organic Pigments*, 4th ed. Weinheim: Wiley-VCH.

[bb24] Kopský, V. & Litvin, D. B. (2010). Editors. *International Tables for Crystallography* Vol. E, *Subperiodic groups*, 2nd ed. Chester: International Union of Crystallography.

[bb25] Laar, B. van & Schenk, H. (2018). *Acta Cryst.* A**74**, 88–92. 10.1107/S2053273317018435 PMC5831587 29493537

[bb47] Lescoeur, H. (1895). *C. R. Acad. Sci.* **121**, 691–692.

[bb46] Liebig, J. (1837). *Ann. Pharm.* **23**, 12–42.

[bb26] Loopstra, B. O. & Rietveld, H. M. (1969). *Acta Cryst.* B**25**, 787–791.

[bb45] Macrae, C. F., Sovago, I., Cottrell, S. J., Galek, P. T. A., McCabe, P., Pidcock, E., Platings, M., Shields, G. P., Stevens, J. S., Towler, M. & Wood, P. A. (2020). *J. Appl. Cryst.* **53**, 226–235. 10.1107/S1600576719014092 PMC6998782 32047413

[bb27] Mehring, M., Berkei, M. & Schürmann, M. (2002). *Z. Anorg. Allg. Chem.* **628**, 1975–1978.

[bb28] Müller, U. (2004). *Z. Anorg. Allg. Chem.* **630**, 1519–1537.

[bb29] Müller, U. (2006). In *Inorganic Structural Chemistry*, 2nd ed., ch. 18. Weinheim: Wiley-VCH.

[bb30] Müller, U. (2012). In *Symmetriebeziehungen zwischen verwandten Kristallstrukturen. Anwendungen der Gruppentheorie in der Kristallchemie*. Wiesbaden: Vieweg+Teubner Verlag. [English translation: *Symmetry Relationships between Crystal Structures* (2013), Oxford University Press.]

[bb31] Nekola, H., Olbrich, F. & Behrens, U. (2002). *Z. Anorg. Allg. Chem.* **628**, 2067–2070.

[bb32] Østreng, E., Sønsteby, H. H., Øien, S., Nilsen, O. & Fjellvåg, H. (2014). *Dalton Trans.* **43**, 16666–16672. 10.1039/c4dt01930j 25265332

[bb33] Ridout, J. & Probert, M. R. (2014). *CrystEngComm*, **16**, 7397–7400.

[bb35] Sheldrick, G. M. (2015*a*). *Acta Cryst.* A**71**, 3–8.

[bb36] Sheldrick, G. M. (2015*b*). *Acta Cryst.* C**71**, 3–8.

[bb37] Wanklyn, J. A. (1869). *Ann. Chem. Pharm.* **150**, 200–206.

[bb38] Wei, C. H. & Hingerty, B. E. (1981). *Acta Cryst.* B**37**, 1992–1997.

[bb39] Weiss, E. (1963). *Helv. Chim. Acta*, **46**, 2051–2054.

[bb40] Weiss, E. (1964). *Z. Anorg. Allg. Chem.* **332**, 197–203.

[bb41] Weiss, E. & Alsdorf, H. (1970). *Z. Anorg. Allg. Chem.* **372**, 206–213.

[bb42] Wheatley, P. J. (1961). *J. Chem. Soc. (London)*, **1961**, 4270–4274.

[bb43] Wondratschek, H. & Müller, U. (2010). *International Tables for Crystallography*, Vol. A1, *Symmetry Relations between Space Groups*, 2nd ed. Chester: International Union of Crystallography.

