# Supplementary material for: Disordered sodium alkoxides from powder data: crystal structures of sodium ethoxide, propoxide, butoxide and pentoxide, and some of their solvates
Source: Acta Crystallogr B Struct Sci Cryst Eng Mater. 2021 Jan 21;77(Pt 1):68–82. doi: 10.1107/S205252062001584X (PMC7941280; doi:10.1107/S205252062001584X)
Supplement: Supplementary file 12 [file b-77-00068-sup12.pdf]

Acta Cryst B

STRUCTURAL SCIENCE
CRYSTAL ENGINEERING
MATERIALS

Volume 77 (2021)

Supporting information for article:

Disordered sodium alkoxides from powder data: Crystal structures of sodium ethoxide, propoxide, butoxide, and pentoxide, and some of their solvates

Maurice Beske, Martin U. Schmidt and Lukas Tapmeyer

Supporting Information

for

"Disordered sodium alkoxides from powder data: Crystal structures of sodium ethoxide, propoxide, butoxide, and pentoxide, and some of their solvates"

by Beske, Schmidt & Tapmeyer

Content of this ESI

1. Syntheses of NaO^{n}Pr, NaO^{n}Bu, and NaO^{n}Am

2. Syntheses of NaO^{n}Pr · 2 ^{n}PrOH NaO^{i}Pr · 5 ^{i}PrOH and NaO^{t}Am · ^{t}AmOH

3. Pre-characterisation

4. Some details on the Rietveld refinement

5. On the crystal structure of solid isopropanol

1. Syntheses of NaO^{n}Pr, NaO^{n}Bu, and NaO^{n}Am

NaO^{n}Pr

0.27 g (12 mmol) of sodium were added to 10 ml (130 mmol) of *n*-propanol (1-propanol) and were allowed to react for 30 minutes at room temperature. The solution was heated to 60 °C and the excess alcohol was removed under vacuum. The resulting solid product was evaporated at 60 °C under vacuum for four hours, yielding a white, phase-pure powder of NaO^{n}Pr.

NaO^{n}Bu

0.23 g (10 mmol) of sodium were added to a solution of 1.0 ml (11 mmol) of *n*-butanol (1-butanol) in 10 ml of toluene. The reaction mixture was heated under reflux for two and a half hours. A slight precipitation of a solid could be observed. Upon cooling to room temperature, the precipitate increased. The excess liquid was removed from the solid with a syringe. The remaining product was evaporated *in vacuo* for one and a half hours at room temperature, yielding a white, phase-pure powder of NaO^{n}Bu.

NaO^{n}Am

0.20 g (8.7 mmol) of sodium were added to 10 ml (92 mmol) of amyl alcohol (1-pentanol). The mixture was heated under reflux for one hour, and the excess alcohol was removed under

vacuum. The resulting solid product was evaporated at 80 °C under vacuum for five hours, producing a white, phase-pure powder of NaO^{n}Am.

2. Syntheses of NaO^{n}Pr · 2 ^{n}PrOH, NaO^{i}Pr · 5 ^{i}PrOH and NaO^{t}Am · ^{t}AmOH

NaO^{n}Pr · 2 ^{n}PrOH

0.29 g (13 mmol) of sodium were added to 3.0 ml (40 mmol) of *n*-propanol (1-propanol). After reacting for 20 minutes at room temperature, a gel containing unreacted sodium was obtained. The mixture was heated to 50 °C for one hour. Afterwards, another 2.0 ml of *n*-propanol were added, and the mixture was heated under reflux for one and a half hour. Finally, a solution was obtained. Upon cooling to room temperature (duration approximately three hours), a gel formed, which contained white, needle-shaped crystals of NaO^{n}Pr · 2 ^{n}PrOH with a size of up to 8 mm.

NaO^{i}Pr · 5 ^{i}PrOH

0.30 g (13 mmol) of sodium were added to 10 ml (130 mmol) of isopropanol (2-propanol). The mixture was allowed to react at room temperature for two hours and subsequently heated under reflux for three hours. After cooling to room temperature, the solution solidified to a wax-like product. The solid was dissolved in a small amount of 2-propanol under reflux leading to a saturated solution. Subsequent cooling to room temperature yielded white, needle-shaped crystals with a size of up to 1 cm.

NaO^{t}Am · ^{t}AmOH

0.21 g (9.1 mmol) of sodium were added to 10 ml (92 mmol) of *tert*-amyl alcohol (2-methyl-2-butanol). The mixture was heated to 50 °C for one and a half hours, then to 70 °C for one hour and finally refluxed for five hours. The obtained solution was cooled to room temperature, yielding white, needle-shaped crystals with a size of up to 1 mm.

3. Pre-characterisation

The stoichiometry of the solvent-free alkoxides was confirmed by decomposition experiments. The powders were reacted with aqueous hydrochloric acid producing the corresponding alcohol and NaCl. The amount of alcohol was determined by ^{1}H-NMR spectroscopy, using a known amount of methanol as an internal standard. The mass of NaCl was determined gravimetrically.
Results:
- NaOEt: 20 mg (0.29 mmol) of NaOEt generated 0.29 mmol of EtOH and 0.31 mmol of NaCl.
- NaO^{n}Pr: 93 mg (1.1 mmol) of NaO^{n}Pr yielded 1.1 mmol of ^{n}PrOH and 1.1 mmol of NaCl.
- NaO^{n}Bu: 94 mg (0.98 mmol) of NaO^{n}Bu delivered 0.98 mmol of ^{n}PrOH and 0.98 mmol of NaCl.
- NaO^{n}Am: 219 mg (2.0 mmol) of NaO^{n}Am generated 8.3 mmol of ^{n}PrOH and 1.9 mmol of NaCl. (Apparently, the sample of NaO^{n}Am contained ^{n}AmOH.)

For the solvates, this analysis could not be performed, because they rapidly decomposed when removed from their alcoholic mother liquor.

4. Some details on the Rietveld refinements

In NaOEt, individual isotropic temperature factors were used for all non-hydrogen atoms. In the other three structures two or three different isotropic temperature factors were used: one for the ordered atoms Na and O, and one or two for the disordered C atoms. H-atom temperature factors were constrained to be 1.2 times the temperature factor of the adjacent carbon atom.

The peak width anisotropy was modelled by spherical harmonics of 6th order, while preventing negative FWHM. Due to the tetragonal symmetry, the inclusion of 6th order harmonics only led to 5 additional parameters and one scale factor.

In the final refinements, no correction for preferred orientation was applied, because all samples were fine powders with a random orientation of the crystallites in the capillaries. A preferred orientation correction using the March-Dollase formalism (Dollase, 1986) in the direction (001) would improve the fit, but the resulting preferred orientation coefficient correlates strongly with the disorder of the alkyl groups in the *xy* plane, instead of describing the preferred orientation of the crystallites in the capillary.

For assessing the correct space groups, Rietveld refinements were performed under strictly identical conditions in $P\ 4/n\ m\ m$ and $P\ \text{-}4\ 2_1\ m$. These conditions were different than in the final refinements. For example, no correction for peak width anisotropy was applied. These differences explain that the *R* values in Tables 1, 3 and 4 are different from those in Table 2.

Reference: Dollase, W. A. (1986). *J. Appl. Cryst.* 19, 267–272.

5. On the crystal structure of solid isopropanol

The following figures show the arrangement of rings and chains in the crystal structures of solid isopropanol:

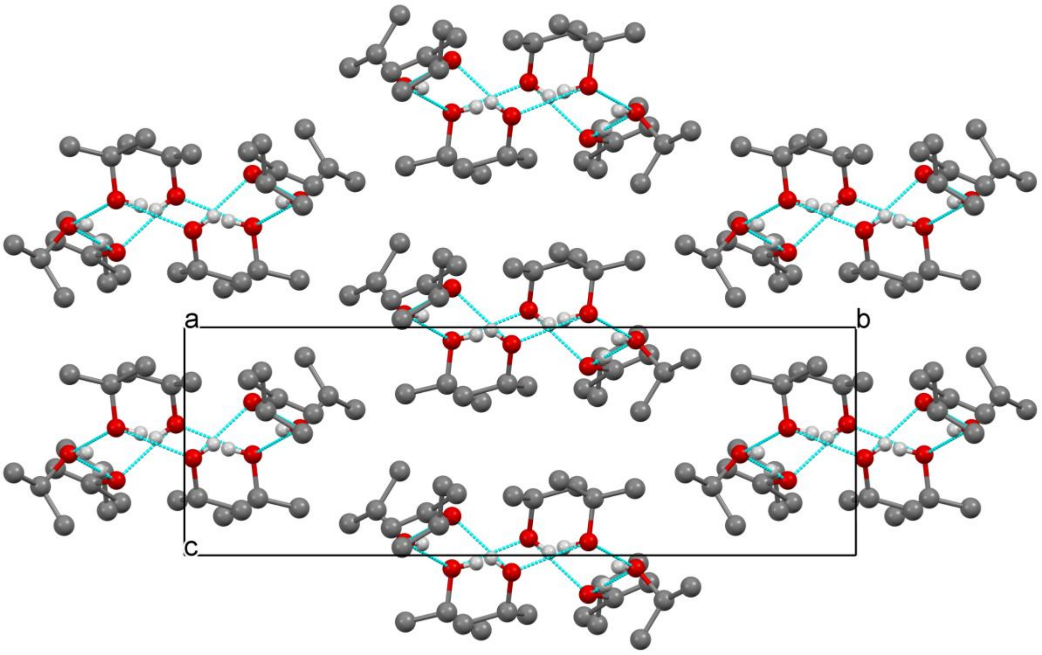

Fig. S1. Crystal structure of solid pure isopropanol: High-pressure phase. The figure shows seven 8-membered rings

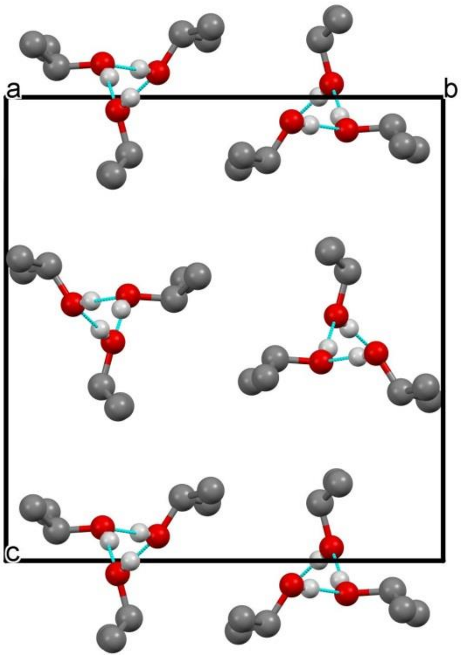

Fig. S2. Crystal structure of solid pure isopropanol: Low-temperature phase, consisting of helices.

Reference for the crystal structure data of solid isopropanol:

Ridout, J. & Probert, M.R. (2014). *CrystEngComm.* 16, 7397.
